# IL‐36*γ* and IL‐36Ra Reciprocally Regulate NSCLC Progression by Modulating GSH Homeostasis and Oxidative Stress‐Induced Cell Death

**DOI:** 10.1002/advs.202101501

**Published:** 2021-08-08

**Authors:** Peng Wang, Wei Yang, Hao Guo, Hong‐Peng Dong, Yu‐Yao Guo, Hu Gan, Zou Wang, Yongbo Cheng, Yu Deng, Shizhe Xie, Xinglou Yang, Dandan Lin, Bo Zhong

**Affiliations:** ^1^ Department of Gastrointestinal Surgery College of Life Sciences Zhongnan Hospital of Wuhan University Wuhan 430071 China; ^2^ Department of Pulmonary and Critical Care Medicine Zhongnan Hospital of Wuhan University Wuhan 430071 China; ^3^ Department of Immunology Medical Research Institute and Frontier Science Center for Immunology and Metabolism Wuhan University Wuhan 430071 China; ^4^ Wuhan Biobank Co., Ltd, Wuhan Wuhan 430075 China; ^5^ Elem Biotech Co., Ltd Wuhan 430075 China; ^6^ Department of Thoracic Surgery Tongji Hospital Tongji Medical College Huazhong University of Science and Technology Wuhan 430030 China; ^7^ CAS Key Laboratory of Special Pathogens Wuhan Institute of Virology Center for Biosafety Mega‐Science Chinese Academy of Sciences Wuhan 430071 China; ^8^ University of Chinese Academy of Sciences Beijing 100049 China; ^9^ Cancer Center Renmin Hospital of Wuhan University Wuhan 430060 China; ^10^ Wuhan Research Center for Infectious Diseases and Cancer Chinese Academy of Medical Sciences Wuhan 430071 China

**Keywords:** glutathione metabolism, IL‐36*γ*; IL‐36Ra, non‐small cell lung cancer, oxidative stress, reactive oxygen species

## Abstract

The balance between antioxidants and reactive oxygen species (ROS) critically regulates tumor initiation and progression. However, whether and how the tumor‐favoring redox status is controlled by cytokine networks remain poorly defined. Here, it is shown that IL‐36*γ* and IL‐36Ra reciprocally regulate the progression of non‐small cell lung cancer (NSCLC) by modulating glutathione metabolism and ROS resolution. Knockout, inhibition, or neutralization of IL‐36*γ* significantly inhibits NSCLC progression and prolongs survival of the *Kras* ^LSL‐G12D/+^ *Tp53* ^fl/fl^ and *Kras* ^LSL‐G12D/+^ *Lkb1* ^fl/fl^ mice after tumor induction, whereas knockout of IL‐36Ra exacerbates tumorigenesis in these NSCLC mouse models and accelerates death of mice. Mechanistically, IL‐36*γ* directly upregulates an array of genes involved in glutathione homeostasis to reduce ROS and prevent oxidative stress‐induced cell death, which is mitigated by IL‐36Ra or IL‐36*γ* neutralizing antibody. Consistently, IL‐36*γ* staining is positively and negatively correlated with glutathione biosynthesis and ROS in human NSCLC tumor biopsies, respectively. These findings highlight essential roles of cytokine networks in redox for tumorigenesis and provide potential therapeutic strategy for NSCLC.

## Graphical abstract

The homeostasis of redox critically regulates tumor initiation and progression. Here, it is shown that IL‐36*γ* and IL‐36Ra reciprocally regulate non‐small cell lung cancer (NSCLC) progression by modulating glutathione metabolism, reactive oxygen species, and oxidative stress‐induced cell death. These findings highlight essential roles of IL‐36 family cytokines in redox for tumorigenesis and potential therapeutic strategy for NSCLC.

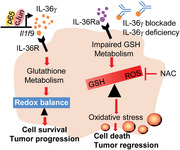

## 1 Introduction

Lung cancer is the most common cause of cancer‐related death worldwide and understanding its biology is crucial for the treatment options of patients and the development of effective therapies.^[^ ^1^, ^2^ ^]^ According to the pathologic features, lung cancer is divided into small cell lung cancer and non‐small cell lung cancer (NSCLC), the latter of which is the major subtype and accounts for about 85% of lung cancer incidences.^[^ ^1^ ^]^ Various somatic mutations in tumor‐driver genes such as *KRAS*, *EGFR*, and *ALK* and tumor suppressor genes such as *TP53* and *LKB1* have been detected in NSCLC tumor tissues.^[^ ^3^, ^4^ ^]^ Studies with genetic mouse models have demonstrated that tumor‐driver genes with gain‐of‐function mutations are sufficient for tumor initiation and progression, which can be accelerated and exacerbated by inactivation of tumor suppressors or introduction of loss‐of‐function tumor suppressors.^[^ ^3^, ^5^, ^6^ ^]^ For example, mutations of KRAS at Gly12 or Gly13 are found in 10–20% NSCLC incidences and mice conditionally expressing Kras^G12D^ in lung spontaneously develop NSCLC.^[^ ^5^ ^]^ *TP53* and *LKB1* are two tumor suppressors and their loss‐of‐function mutations are commonly found in *KRAS*‐mutated NSCLC patients.^[^ ^7^, ^8^, ^9^, ^10^ ^]^ The progression of NSCLC in *Kras* ^G12D^ mice is substantially accelerated and aggravated by inactivation of *Tp53* (*Kras* ^LSL−G12D/+^ *Tp53* ^fl/fl^; KP) or *Lkb1* (*Kras* ^LSL−G12D/+^ *Lkb1* ^fl/fl^; KL).^[^ ^6^, ^11^ ^]^ Accordingly, these mutations represent attractive targets for therapeutic intervention and the genetic mouse models provide powerful tools for the screen and evaluation of effective therapies for NSCLC.^[^ ^12^, ^13^, ^14^, ^15^ ^]^

Oxidative stress is caused by imbalanced reactive oxygen species (ROS) and antioxidants and has been implicated in the pathology of many diseases including cancer.^[^ ^16^ ^]^ ROS are generated during aerobic metabolism and hyperproliferation of cancer cells is accompanied by high levels of ROS.^[^ ^17^ ^]^ ROS react with lipids, proteins and nucleic acids, leading to the formation of oxidized substances such as the membrane lipid peroxidation, and higher levels of ROS than thresholds can cause senescence and cell death. Malignant cells alleviate the oxidative stress by increasing the antioxidants to neutralize excessive ROS and counteract the cytotoxic damages.^[^ ^18^ ^]^ Consistent with this notion, recent multiomics studies of NSCLC have revealed that genes involved in oxidant response are frequently mutated and that pathways involved in antioxidants metabolism are upregulated.^[^ ^8^, ^9^, ^10^ ^]^ In addition, it has been shown that *Kras* ^G12D^ stimulates transcription of endogenous antioxidant genes to promote NSCLC development, which can be accelerated by supplementation of *N*‐acetylcysteine (NAC) or vitamin E in the drinking water,^[^ ^19^, ^20^ ^]^ suggesting that increasing the amounts of antioxidants to decrease ROS is essential for NSCLC progression.

Glutathione (l‐*γ*‐glutamyl‐l‐cysteinyl‐glycine, GSH) is a non‐enzymatic antioxidant that plays central roles in countering oxidative stress. The homeostasis of GSH is achieved through de novo synthesis and salvage pathways that involve a series of sequential enzymatic reactions.^[^ ^17^ ^]^ The biosynthesis of GSH depends on glutamate cysteine ligase (GCL)‐mediated synthesis of *γ*‐glutamylcysteine from l‐glutamate and cysteine, and glutathione synthetase (GSS)‐mediated addition of glycine to the C‐terminal of *γ*‐glutamylcysteine, in which GCL catalyzes the initial rate‐limiting step and GSS the second and final step.^[^ ^21^ ^]^ Glutathione peroxidases (GPXs) transform GSH into oxidized disulfide form glutathione disulfide (GSSG) while simultaneously reduce ROS levels.^[^ ^22^ ^]^ Sequentially, glutathione reductase (GSR) transforms GSSG into GSH by using reduced nicotinamide adenine dinucleotide phosphate (NADPH) as the electron donor.^[^ ^23^ ^]^ In the process, NADPH is oxidized to NADP^+^ which is transformed into NADPH by glucose‐6‐phosphate dehydrogenase (G6PD) and 6‐phosphogluconate dehydrogenase (6PGD) involved in the pentose phosphate pathway.^[^ ^24^, ^25^ ^]^ It has been observed that GSH levels are elevated in NSCLC tumor tissues and high levels of GSH are associated with poor prognosis after surgery and with resistance to chemo‐ and radio‐therapies.^[^ ^26^, ^27^, ^28^ ^]^ Therefore, targeting GSH homeostasis would provide plausible therapeutic intervention for NSCLC.

IL‐36 belongs to the IL‐1 family of cytokines, including IL‐36*α*, IL‐36*β*, and IL‐36*γ* that bind to IL‐36 receptor (IL‐36R) to trigger signaling cascades in a manner dependent on MyD88.^[^ ^29^, ^30^, ^31^ ^]^ IL‐36R signaling is involved in skin inflammation by promoting IL‐17A production in *γδ*TCR^+^ cells and CD4^+^ T cells and type I IFN in plasmacytoid dendritic cells and in colitis by modulating IL‐22 production and mucosal repair.^[^ ^32^, ^33^, ^34^, ^35^, ^36^, ^37^ ^]^ IL‐36Ra is a natural antagonist of IL‐36R and binds IL‐36R with higher affinity than the IL‐36 agonistic cytokines.^[^ ^38^ ^]^ Humans carrying mutations in *IL1F5* (encoding IL‐36Ra) develop generalized pustular psoriasis (GPP), suggesting crucial roles of imbalanced IL‐36 signaling in skin inflammation.^[^ ^39^, ^40^ ^]^ Whether IL‐36R signaling regulates inflammation‐independent biological process is unknown. In this study, we demonstrate that IL‐36*γ* and IL‐36Ra reciprocally regulate GSH homeostasis during NSCLC progression in a manner independently of its regulation of inflammatory cytokine and chemokine production. Knockout, inhibition or neutralization of IL‐36*γ* significantly inhibits NSCLC progression and prolongs survival of the *Kras* ^LSL‐G12D/+^ *Tp53* ^fl/fl^ and *Kras* ^LSL‐G12D/+^ *Lkb1* ^fl/fl^ mice after tumor induction, reduces GSH levels and increases oxidative stress in the tumors and promotes tumor cell death, whereas knockout of IL‐36Ra has opposite effects. In addition, IL‐36*γ* staining is positively and negatively correlated with glutathione biosynthesis and ROS in human NSCLC tumor biopsies, respectively. These findings highlight the role of IL‐36R signaling in GSH homeostasis during NSCLC progression and suggest IL‐36*γ* as a therapeutic target for NSCLC.

## 2 Results

### 2.1 IL‐36*γ* is Highly Expressed in NSCLC Tumor Tissues

Interleukin family cytokines play essential roles in inflammation that has been implicated in the initiation and development of lung cancer.^[^ ^41^ ^]^ In an attempt to screen cytokines and chemokines that were differentially expressed in NSCLC tumor tissues versus normal lung tissues,^[^ ^42^, ^43^ ^]^ we identified that *IL1F9* (encoding IL‐36*γ*, an IL‐36 cytokine member) but not *IL1F6* or *IL1F8* (encoding IL‐36*α* or IL‐36*β*, respectively) was highly expressed in the tumor tissues compared to the normal tissues (Cohort 1) (Figure S1A and Table S1, Supporting Information). This observation was confirmed with another independent cohort of tumors and normal lung tissues from NSCLC patients (Cohort 2) (Figure S1B and Table S2, Supporting Information), and was consistent with the data from the TCGA database (Figure S1C and Table S3, Supporting Information). In addition, the levels of mouse *Il1f9* were upregulated in the tumor‐burdened lungs from *Kras* ^LSL‐G12D/+^ *Lkb1* ^fl/fl^ mice that were intranasally injected with Ad‐Cre (Figure S1D, Supporting Information). Analysis of mouse *Il1f9* gene promoter identified multiple cis‐regulatory elements that were recognized by various transcription factors, including NF‐*κ*B, Fos‐Jun and STAT3 (Figure S1E,F, Supporting Information). Results from chromatin immunoprecipitation (ChIP) assays showed increased binding of p65 and cJun but not (p)STAT3 on the promoter of *Il1f9* gene in the tumor tissues compared with the normal lung tissues from *Kras* ^LSL‐G12D/+^ *Lkb1* ^fl/fl^ mice that were intranasally injected with Ad‐Cre or Ad‐Vec (Figure S1E,F, Supporting Information). Collectively, these data indicate that p65 and cJun mediate upregulation of IL‐36*γ* in tumor tissues during NSCLC progression.

### 2.2 Knockout of IL‐36*γ* and IL‐36Ra Reciprocally Regulates NSCLC Progression

Single‐cell mRNA transcriptome analysis revealed that *Il1f9* was primarily expressed in neutrophils, *Il1f5* (encoding IL‐36Ra) was highly expressed in endothelial cells, neutrophils, and Lyz2^+^ mono‐macrophages/dendritic cells (mono‐*ϕ*/DCs), and *Il1rl2* (encoding IL‐36R) was expressed in endothelial cells, epithelial tumor cells, and neutrophils in lung tumors from KL mice (Figure S2A–E, Supporting Information). In contrast, *Il1f6* and *Il1f8* were undetectable (Figure S2D, Supporting Information), indicating that IL‐36R signaling is primarily modulated by IL‐36*γ* and IL‐36Ra during NSCLC progression.

**Figure 1:**
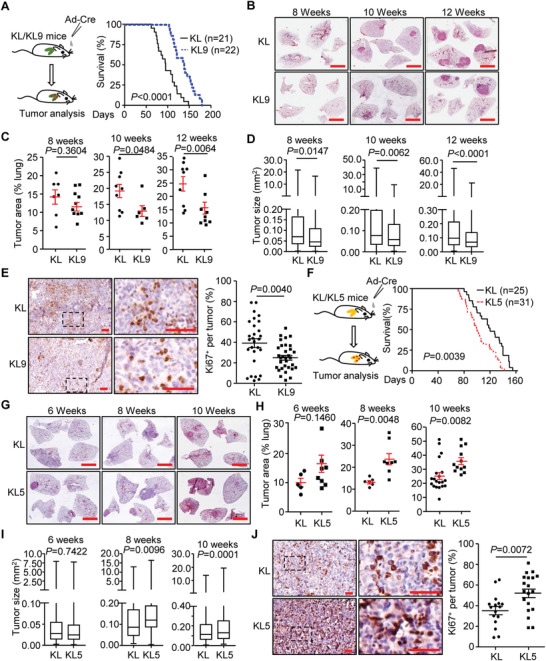
IL‐36*γ* promotes and IL‐36Ra inhibits NSCLC progression of the KL mouse model. A) A scheme of tumor induction of *Kras* ^LSL‐G12D/+^ *Lkb1* ^fl/fl^ (KL) and *Kras* ^LSL‐G12D/+^ *Lkb1* ^fl/fl^ *Il1f9* ^−/‐^ (KL9) mice (left). Survival of KL (*n* = 21) and KL9 (*n* = 22) mice that were intranasally injected with Ad‐Cre (2 × 10^6^ pfu) (right). B) Images of HE staining of tumor‐burdened lungs of KL and KL9 mice that were intranasally injected with Ad‐Cre for 8, 10, or 12 weeks, respectively. C) Tumor burden in the lungs of KL and KL9 mice that were injected with Ad‐Cre for 8 weeks (*n* = 7 and 10 mice for KL and KL9, respectively), 10 weeks (*n* = 9 and 6 mice for KL and KL9, respectively), or 12 weeks (*n* = 9 and 9 mice for KL and KL9, respectively). D) Box plot of individual tumor size in the lungs of KL and KL9 mice that were intranasally injected with Ad‐Cre for 8, 10, or 12 weeks, respectively. E) Images (left) and quantification analysis (right) of Ki67 staining in individual lung tumors from KL (*n* = 28) and KL9 (*n* = 32) mice that were intranasally injected with Ad‐Cre for 10 weeks. F) A scheme of tumor induction of KL and *Kras* ^LSL‐G12D/+^ *Lkb1* ^fl/fl^ *Il1f5* ^−/−^(KL5) mice (left). Survival of KL (*n* = 25) and KL5 (*n* = 31) mice that were intranasally injected with Ad‐Cre (2 × 10^6^ pfu) (right). G) Images of HE staining of tumor‐burdened lungs of KL and KL5 mice that were intranasally injected with Ad‐Cre for 6, 8, or 10 weeks, respectively. (H) Tumor burden in the lungs of KL and KL5 mice that were intranasally injected with Ad‐Cre for 6 weeks (*n* = 5 and 8 mice for KL and KL5, respectively), 8 weeks (*n* = 6 and 8 mice for KL and KL5, respectively) or 10 weeks (*n* = 20 and 12 mice for KL and KL5, respectively). I) Box plot of individual tumor size in the lungs of KL and KL5 that were intranasally injected with Ad‐Cre for 6, 8, or 10 weeks, respectively. J) Images (left) and quantification analysis (right) of Ki67 staining in individual lung tumors from KL (*n* = 16) and KL5 (*n* = 19) mice that were intranasally injected with Ad‐Cre for 8 weeks. Graphs show mean ± SEM (C–E, H–J). Two‐tailed student's *t*‐test (C–E, H–J) or Log‐Rank analysis (A,F). Scale bars represent 5 mm (B,G) or 50 µm (E,J), respectively. Data are combined results of three independent experiments (A,F) or representative results of two independent experiments (B–E,G–J).

**Figure 2:**
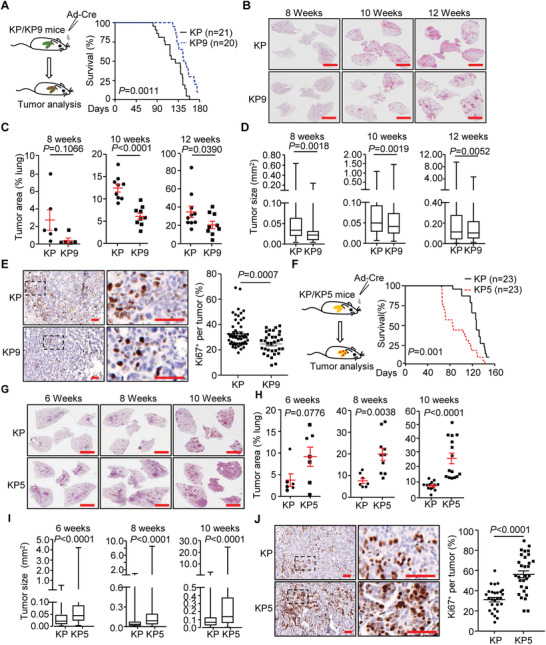
IL‐36*γ* promotes and IL‐36Ra inhibits NSCLC progression of the KP mouse model. A) A scheme of tumor induction of *Kras* ^LSL‐G12D/+^ *Tp53* ^fl/fl^ (KP) and *Kras* ^LSL‐G12D/+^ *Tp53* ^fl/fl^ *Il1f9* ^−/‐^ (KP9) mice (left). Survival of KP (*n* = 21) and KP9 (*n* = 20) mice that were intranasally injected with Ad‐Cre (2 × 10^6^ pfu) (right). B) Images of HE staining of tumor‐burdened lungs of KP and KP9 mice after intranasal injection with Ad‐Cre for 8, 10, or 12 weeks, respectively. C) Tumor burden in the lungs of KP and KP9 mice that were intranasally injected with Ad‐Cre for 8 weeks (*n* = 6 and 5 mice for KP and KP9, respectively), 10 weeks (*n* = 9 and 9 mice for KP and KP9, respectively), or 12 weeks (*n* = 10 and 9 mice for KP and KP9, respectively). D) Box plot of individual tumor size in the lungs of KP and KP9 mice that were intranasally injected with Ad‐Cre for 8, 10, or 12 weeks, respectively. E) Images (left) and quantification analysis (right) of Ki67 staining in individual lung tumors from KP (*n* = 53) and KP9 (*n* = 33) mice that were intranasally injected with Ad‐Cre for 10 weeks. F) A scheme of tumor induction of KP and *Kras* ^LSL‐G12D/+^ *Tp53* ^fl/fl^ *Il1f5* ^−/−^(KP5) mice (left). Survival of KP (*n* = 23) and KP5 (*n* = 23) mice that were intranasally injected with Ad‐Cre (2 × 10^6^ pfu) (right). G) Images of HE staining of tumor‐burdened lungs of KP and KP5 mice after intranasal injection with Ad‐Cre for 6, 8, or 10 weeks, respectively. H) Tumor burden in the lungs of KP and KP5 mice that were intranasally injected with Ad‐Cre for 6 weeks (*n* = 6 and 7 mice for KP and KP5, respectively), 8 weeks (*n* = 7 and 10 mice for KP and KP5, respectively), or 10 weeks (*n* = 13 and 15 mice for KP and KP5, respectively). I) Box plot of individual tumor size in the lungs of KP and KP5 that were intranasally injected with Ad‐Cre for 6, 8, or 10 weeks, respectively. J) Images (left) and quantification analysis (right) of Ki67 staining in individual lung tumors from KP (*n* = 27) or KP5 (*n* = 30) mice that were intranasally injected with Ad‐Cre for 8 weeks. Graphs show mean ± SEM (C–E,H–J).Two‐tailed student's *t*‐test (C–E,H–J) or Log‐Rank analysis (A,F). Scale bars represent 5 mm (B,G) or 50 µm (E,J), respectively. Data are combined results of three independent experiments (A,F) or representative results of two independent experiments (B–E,G–J).

We next investigated the role of IL‐36*γ* in NSCLC development with the KL mouse model. As shown in **Figure** **1**A, knockout of IL‐36*γ* significantly prolonged the survival of KL mice that were intranasally injected with Ad‐Cre (median overall survival 138 days vs 95 days, *p *< 0.0001) (Figure 1A). Histological analysis revealed that the tumor areas and sizes were significantly smaller in lungs of *Kras* ^LSL‐G12D/+^ *Lkb1* ^fl/fl^ *Il1f9* ^−/−^ (KL9) mice than in the lungs of KL mice after tumor induction (Figure 1B–D). Consistently, the Ki67 staining in the lung tumors of KL9 mice was significantly lower than that from KL mice (Figure 1E). Considering that IL‐36*γ* and IL‐36Ra are the major IL‐36 cytokines in the lung tumors and that IL‐36Ra antagonizes IL‐36*γ*‐triggered signaling by competitively binding to IL‐36R (Figure S2D,E, Supporting Information),^[^ ^38^ ^]^ we reasoned that IL‐36Ra might antagonize the effects of IL‐36*γ* on promotion of NSCLC progression. Expectedly, knockout of IL‐36Ra accelerated death and promoted tumor progression in the lungs of KL mice after tumor induction (Figure 1F–I). Consistently, the Ki67 staining was significantly elevated in the lung tumors from KL5 mice compared to KL mice (Figure 1J). Similarly, in the *Kras* ^LSL‐G12D/+^ *Tp53* ^fl/fl^ (KP) NSCLC mouse model, knockout of IL‐36*γ* prolonged the survival, inhibited tumor progression and impaired cell proliferation in the lung tumors of the KP mice after intranasal injection of Ad‐Cre (**Figure** **2**A–E). Conversely, knockout of IL‐36Ra accelerated death, promoted tumor progression and enhanced cell proliferation in the lung tumors of KP mice after tumor induction (Figure 2F–J). These data collectively demonstrate that IL‐36*γ* promotes and IL‐36Ra reciprocally suppresses tumor progression in NSCLC mouse models, respectively.

### 2.3 IL‐36*γ* and IL‐36Ra Modulate GSH Homeostasis and Oxidative Stress in NSCLC Tumor

Transcriptome analysis of lung tumors from KL, KL5, and KL9 mice suggested that genes differentially expressed in KL9 versus KL tumors and in KL5 versus KL tumors were co‐enriched in cytokine and chemokine signaling pathways and glutathione (GSH) metabolism pathways (Figure S3A and Table S4, Supporting Information). Unexpectedly, however, gene‐set enrichment analysis (GSEA) suggested that cytokine and chemokine signaling pathways were similarly downregulated in tumors from KL9 and KL5 mice compared to tumors from KL mice (Figure S3B–E and Table S5, Supporting Information), which was confirmed by quantitative real‐time PCR (qRT‐PCR) analysis (Figure S3F,G, Supporting Information). In the KP mouse model, cytokine and chemokine signaling pathways were similarly downregulated in IL‐36*γ* or IL‐36Ra deficient tumors compared to the controls (Figure S3H–M and Tables S4, S5, Supporting Information). These data suggest that the cytokine and chemokine expression or signaling is unlikely responsible for IL‐36*γ*‐ and IL‐36Ra‐mediated reciprocal regulation of NSCLC progression.

It has been reported that overexpression of IL‐36*γ* in tumor cells in synergy with IL‐12 promotes type I immune responses to promote tumor regression and that IL‐36*γ* promotes IL‐17 production by *γδ*TCR^+^ cells and CD4^+^ T cells in skin inflammation.^[^ ^32^, ^44^ ^]^ However, knockout of IL‐36*γ* did not affect lymphocyte infiltration in tumor‐burdened lungs in the KL mouse model (Figure S4A, Supporting Information). The percentages and numbers of CD8^+^IFN*γ* ^+^, CD4^+^IFN*γ* ^+^, CD4^+^IL‐17A^+^, or *γδ*TCR^+^IL‐17A^+^ cells in tumor‐burdened lungs or bronchial draining lymph nodes (dLNs) were comparable between KL and KL9 mice after tumor induction (Figure S4B–F, Supporting Information), indicating that IL‐36*γ* regulates NSCLC progression independently of lymphocyte differentiation or expansion in vivo. In this context, our single‐cell mRNA sequncing (scRNA‐seq) data suggested that lymphocytes in the tumor‐burdened lungs barely expressed IL‐36R (Figure S2E, Supporting Information).

**Figure 3:**
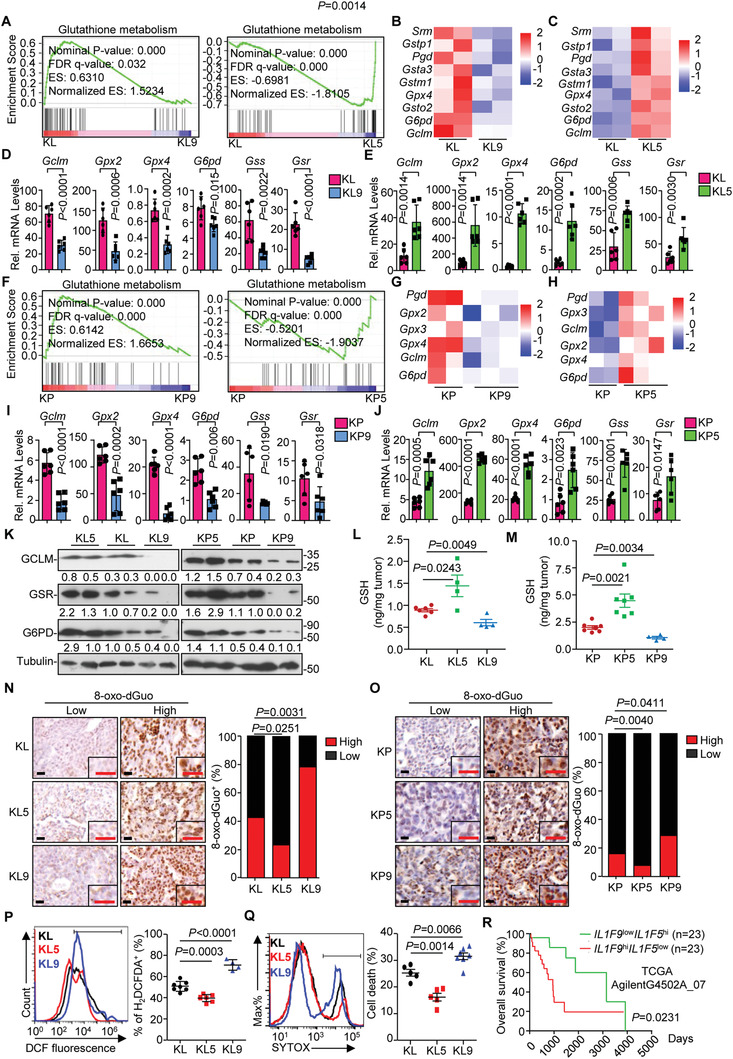
IL‐36*γ* and IL‐36Ra reciprocally regulate glutathione homeostasis and ROS levels during NSCLC progression. A–C) GSEA plot of the glutathione metabolism pathway (A) and z‐score heatmap of the indicated genes from the transcriptome data of lung tumors from KL (*n* = 2) and KL9 (*n* = 2) (B) or KL (*n* = 2) and KL5 (*n* = 2) (C) mice that were intranasally injected with Ad‐Cre for 10 weeks or 8 weeks, respectively. D–E) Quantitative real‐time PCR (qRT‐PCR) analysis of the signature genes for GSH homeostasis in lung tumors isolated from KL (*n* = 6) and KL9 (*n* = 6) mice that were intranasally injected with Ad‐Cre for 10 weeks (D) or from KL (*n* = 6) and KL5 (*n* = 6) mice that were injected with Ad‐Cre for 8 weeks (E). F–H) GSEA plot of the glutathione metabolism pathway (F) and z‐score heatmap of the indicated genes from the transcriptome analysis of lung tumors from KP (*n* = 2) and KP9 (*n* = 3) (G) or KP (*n* = 2) and KP5 (*n* = 3) (H) mice that were intranasally injected with Ad‐Cre for 10 weeks or 8 weeks, respectively. I–J) qRT‐PCR analysis of the signature genes for GSH metabolism in the lung tumors of KP (*n* = 6) and KP9 (*n* = 6) mice that were that were intranasally injected with Ad‐Cre for 10 weeks (I) or from KP (*n* = 6) and KP5 (*n* = 6) mice that were intranasally injected with Ad‐Cre for 8 weeks (J). K) Immunoblot analysis of the signature proteins for GSH homeostasis in lung tumors from KL (*n* = 2), KL5 (*n* = 2), and KL9 (*n* = 2) (left), or KP (*n* = 2), KP5 (*n* = 2), and KP9 (*n* = 2) (right) mice that were intranasally injected with Ad‐Cre for 10 weeks. L–M) GSH levels in the lung tumors from KL (*n* = 6), KL5 (*n* = 4), and KL9 (*n* = 4) mice (L) or KP (*n* = 7), KP5 (*n* = 7), and KP9 (*n* = 4) mice (M) that were were intranasally injected with Ad‐Cre for 10 weeks. M–O) Images (left) and quantification analysis (right) of 8‐oxo‐dGuo staining in the lung tumors from KL (*n* = 7), KL5 (*n* = 6), and KL9 (*n* = 4) mice (N) or KP (*n* = 6), KP5 (*n* = 5), and KP9 (*n* = 5) mice (O) that were intranasally injected with Ad‐Cre for 10 weeks. P) Flow cytometry (left) and quantification analysis (right) of H_2_DCFDA staining of single‐cell suspensions of lung tumors from KL (*n* = 7), KL5 (*n* = 6), and KL9 (*n* = 4) mice that were intranasally injected with Ad‐Cre for 10 weeks. Q) Flow cytometry (left) and quantification analysis (right) of SYTOX Green staining of single‐cell suspensions of lung tumors from KL (*n* = 5), KL5 (*n* = 5), and KL9 (*n* = 7) mice that were intranasally injected with Ad‐Cre for 10 weeks. R) Kaplan–Meier survival curves pf NSCLC patients with *IL1F9* ^high^ *IL1F5* ^low^ (*n* = 23) and *IL1F9* ^low^ *IL1F5* ^high^ low (*n* = 23) expression pattern (TCGA AgilentG4502A_07). Graphs show mean ± SEM (D,E,I,J,L,M,P,Q). Two‐tailed student's *t*‐test (D,E,I,J,L–Q) or Log‐Rank analysis (*R*). Scale bars represent 50 µm (N,O). Data are representative results of two independent experiments (D,E,L–Q).

Further analysis of the transcriptome data suggested that genes involved in GSH metabolism were downregulated in lung tumors from KL9 or KP9 mice and upregulated in lung tumors from KL5 or KP5 mice compared to the respective controls (**Figure** **3**A–C,F–H and Tables S4, S5, Supporting Information). Results from qRT‐PCR and immunoblot analyses confirmed that the levels of *Gclm*, *Gpx2/4*, *Gss*, *Gsr* and *G6pd* mRNA and the levels of GCLM, GSR, and G6PD were decreased and upregulated in IL‐36*γ*‐ and IL‐36Ra‐deficient lung tumors compared to the controls, respectively (Figure 3D–E,I–K). These proteins are key enzymes involved in GSH biosynthesis or regeneration.^[^ ^21^, ^22^, ^23^, ^24^ ^]^ Consistently, the levels of GSH were increased in lung tumors from KL5 or KP5 mice and decreased in lung tumors from KL9 or KP9 mice compared to the controls, respectively (Figure 3L–M). In contrast, the GSSG levels and the ratios of NADPH/NADP^+^ were decreased in lung tumors from KL5 or KP5 mice and increased in lung tumors from KL9 or KP9 mice compared to the controls, respectively (Figure S5A,B, Supporting Information). GSH is an antioxidant essential for neutralization of ROS and prevention of aberrant lipid or DNA oxidation.^[^ ^17^ ^]^ Interestingly, the staining of 8‐oxo‐7,8‐dihydro‐2′‐deoxyguanosine (8‐oxo‐dGuo) (a biomarker of oxidative DNA damage) or 4‐Hydroxynonenal (4HNE) (a lipid‐peroxidation maker) were significantly higher in lung tumors from KL9 or KP9 mice and lower in lung tumors from KL5 or KP5 mice than the controls, respectively (Figure 3N,O and Figure S5C,D, Supporting Information). In addition, the cytosolic ROS level was significantly increased in lung tumors from KL9 mice and decreased in lung tumors from KL5 mice compared to the controls, as indicated by the staining with the fluorescent probe H_2_DCFDA, a cytosolic ROS sensor (Figure 3P), indicating that knockout of IL‐36*γ* and IL‐36Ra aggravates and alleviates oxidative stress in NSCLC tumors, respectively. Consistently, the percentages of dead cells were higher and lower in IL‐36*γ*‐ and IL‐36Ra‐deficeint tumors compared to the controls, respectively, as indicated by staining with the SYTOX Green fluorescent probe (Figure 3Q), indicating that cells in tumor tissues are prone to death by IL‐36*γ* deficiency or inhibition of IL‐36R signaling. Consistently with this notion, IL‐36*γ* staining was negatively and positively correlated with 8‐oxo‐dGuo and GCLM staining in human NSCLC tumor biopsies, respectively (Figure S5E,F and Table S6, Supporting Information), and the expression pattern of *IL1F9* ^hi^ *IL1F5* ^low^ in tumor predicted poor prognosis of NSCLC patients (Figure 3R and Table S7, Supporting Information). Together, these data suggest that IL‐36*γ* and IL‐36Ra reciprocally regulate cell death by modulating GSH homeostasis and oxidative stress during NSCLC progression.

### 2.4 Antioxidant Accelerates Tumor Development in IL‐36*γ*‐Deficient KL Mice

**Figure 4:**
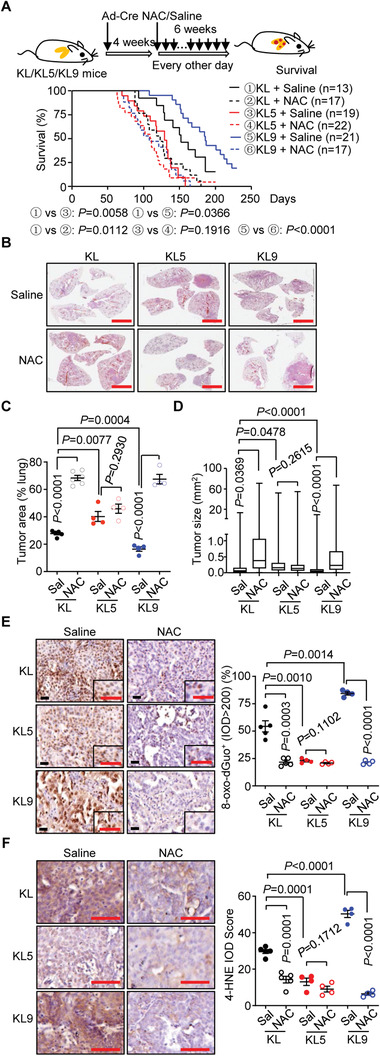
NAC accelerates tumor development in KL and KL9 mice but not KL5 mice. A) A scheme of tumor induction of KL, KL5, or KL9 mice that were intranasally injected with Ad‐Cre for 4 weeks followed by intraperitoneal injection of saline or NAC (5 mg in 200 µL saline) every other day for additional 6 weeks (upper). Survival (lower) of KL (*n* = 13 and 17 mice for saline and NAC, respectively), KL5 (*n* = 19 and 22 mice for saline and NAC, respectively), or KL9 (*n* = 21 and 17 mice for saline and NAC, respectively) mice treated as in the scheme. B) Images of HE staining of tumor‐burdened lungs from KL, KL5, or KL9 mice treated as in (A). C,D) Tumor burden (C) and tumor size (D) of KL (*n* = 5 and 6 mice for saline and NAC, respectively), KL5 (*n* = 4 and 4 mice for saline and NAC, respectively), or KL9 (*n* = 4 and 4 mice for saline and NAC, respectively) mice treated as in (A). E) Images (left) and quantification analysis (right) of 8‐oxo‐dGuo staining in the lung tumors from KL (*n* = 5 and 5 mice for saline and NAC, respectively), KL5 (*n* = 4 and 4 mice for saline and NAC, respectively) or KL9 (*n* = 4 and 4 mice for saline and NAC, respectively) mice treated as in (A). F) Images (left) and quantification analysis (right) of 4HNE staining in the lung tumors from KL (*n* = 5 and 5 mice for saline and NAC, respectively), KL5 (*n* = 4 and 4 mice for saline and NAC, respectively) or KL9 (*n* = 4 and 4 mice for saline and NAC, respectively) mice treated as in (A). Graphs show mean ± SEM (C–F). Two‐tailed student's *t*‐test (C–F) or Log‐Rank analysis (A). Scale bars represent 5 mm (B) or 50 µm (E, F). Data are combined results of three independent experiments (A) or representative results of two independent experiments (B–F).

*N*‐acetylcysteine (NAC) is a powerful antioxidant that accelerates lung cancer progression in mice by promoting ROS resolution and alleviating oxidative stress.^[^ ^19^ ^]^ We next examined the effect of NAC on IL‐36*γ*‐ and IL‐36Ra‐mediated regulation of NSCLC progression. The results suggested that treatment of NAC significantly accelerated death and tumor development of KL or KL9 mice but not KL5 mice after tumor induction (**Figure** **4**A–C). In addition, treatment of NAC accelerated tumor development and mouse death in KL or KL9 mice to an extent similar to KL5 mice that were received saline or NAC after tumor induction (Figure 4A–D), suggesting that antioxidant abolishes the protection against NSCLC progression by IL‐36*γ* deficiency. Consistently, the 8‐oxo‐dGuo or 4HNE staining was decreased in lung tumors from NAC‐treated KL or KL9 mice but not saline‐ or NAC‐treated KL5 mice, and the 8‐oxo‐dGuo or 4HNE staining was similar among NAC‐treated KL or KL9 tumors and saline‐ or NAC‐treated KL5 tumors (Figure 4E,F). Collectively, these data further support the notion that IL‐36*γ* and IL‐36Ra reciprocally regulate NSCLC progression primarily through modulating the ROS and oxidative stress.

### 2.5 IL‐36*γ* Promotes GSH Biogenesis and Protects against Oxidative Stress‐Induced Cell Death

**Figure 5:**
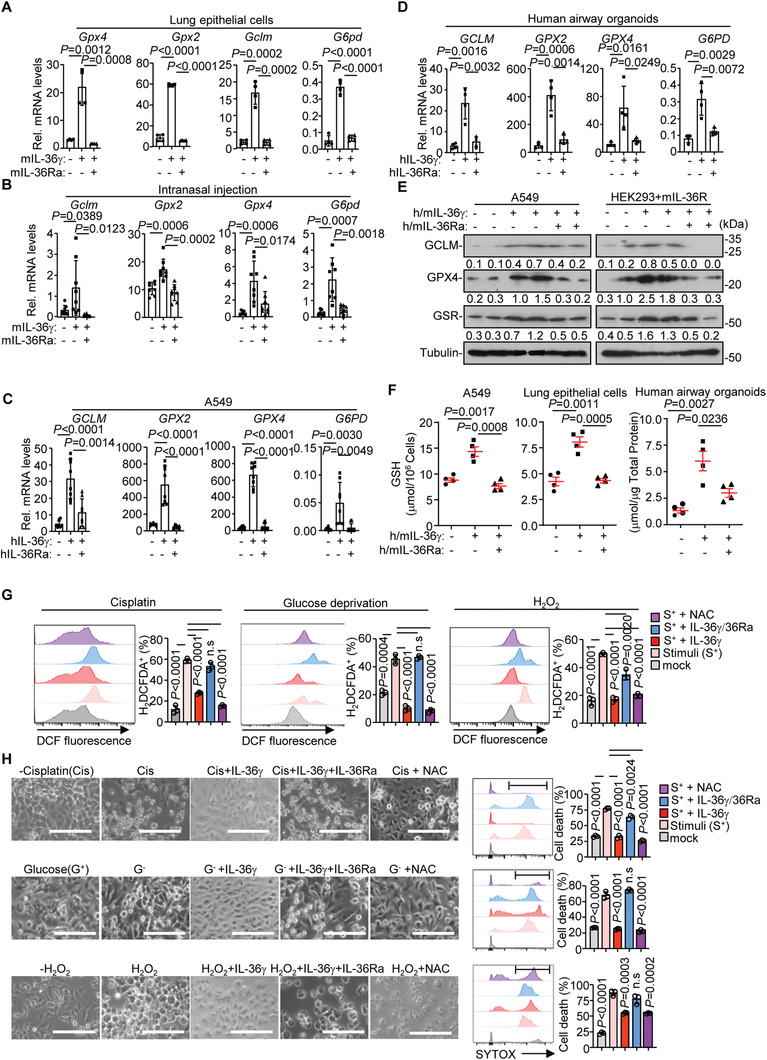
IL‐36*γ* promotes GSH biogenesis and protects against oxidative stress‐induced cell death. A) qRT‐PCR analysis of *Gclm*, *Gpx2*, *Gpx4*, and *G6pd* in primary mouse lung epithelial cells (*n* = 4 technical replicates) that were unstimulated or stimulated with IL‐36*γ* (20 ng mL^−1^) in the presence or absence of IL‐36Ra (20 ng mL^−1^) for 6 h. B) qRT‐PCR analysis of *Gclm*, *Gpx2*, *Gpx4*, and *G6pd* in lungs from C57BL/6 mice that were intranasally injected with PBS (*n* = 8, 50 µL), IL‐36*γ* (*n* = 8, 0.5 µg in 50 µL PBS), or IL‐36*γ* plus IL‐36Ra (*n* = 8, 0.5 µg IL‐36*γ*, and 0.5 µg IL‐36Ra in 50 µL PBS) for 24 h. C,D) qRT‐PCR analysis of *Gclm*, *Gpx2*, *Gpx4*, and *G6pd* in A549 cells (C) (*n* = 4 technical replicates) or in human lung organoid (D) (*n* = 4 technical replicates) that were unstimulated or stimulated with IL‐36*γ* (20 ng mL^−1^) in the presence or absence of IL‐36Ra (20 ng mL^−1^) for 6 h. E) Immunoblot analysis of *Gclm*, *Gpx4*, and *Gsr* in A549 cells (left) or HEK293‐mIL‐36R cells (right) that were unstimulated or stimulated with human or mouse IL‐36*γ* (20 ng mL^−1^) in the presence or absence of human or mouse IL‐36Ra (20 ng mL^−1^) for 8 h. F) GSH levels in in A549 cells (left), mouse lung epithelial cells (middle), or human lung organoids (right) (*n* = 4 technical replicates) that were unstimulated or stimulated with h/mIL‐36*γ* (20 ng mL^−1^) in the presence or absence of h/mIL‐36Ra (20 ng mL^−1^) for 8 h. G) Flow cytometry and quantification analysis of H_2_DCFDA staining in A549 cells that were treated with cisplatin (Cis, 20 µm) for 12 h followed by stimulation with IL‐36*γ* (20 ng mL^−1^), IL‐36*γ* plus IL‐36Ra (20 ng mL^−1^), or IL‐36*γ* plus NAC (5 mm) for 8 h (left), cultured in glucose‐free DMEM (G^−^) for 16 h followed by stimulation with IL‐36*γ* (20 ng mL^−1^), IL‐36*γ* plus IL‐36Ra (20 ng mL^−1^), or IL‐36*γ* plus NAC (5 mm) for 8 h (middle), or stimulated with IL‐36*γ* (20 ng mL^−1^), IL‐36*γ* plus IL‐36Ra (20 ng mL^−1^), or IL‐36*γ* plus NAC (5 mm) for 7 h followed by H_2_O_2_ (16 mm) treatment for 1 h (right) (*n* = 3 technical replicates). H) Images (left) and flow cytometry and quantification analysis of SYTOX Green dead cell (right) of A549 cells treated as in (G) (*n* = 3 technical replicates). Graphs show mean ± SEM (A–D,F–H). Two‐tailed student's *t*‐test (A–D,F–H). Scale bars represent 100 µm (H). Data are representative results of two independent experiments (A–H).

Because the GSH levels were increased and decreased in IL‐36Ra‐ and IL‐36*γ*‐deficient tumors respectively, we next examined whether IL‐36*γ* and IL‐36Ra directly regulated GSH biogenesis. As shown in **Figure** **5**A,B, mIL‐36*γ* induced expression of *Gclm*, *G6pd*, and *Gpxs* that encoded proteins involved in GSH biogenesis in lung epithelial cells and in the lungs of mice but not in alveolar macrophages, and such an induction was compromised by mIL‐36Ra (Figure 5A,B and Figure S6A,B, Supporting Information). These observations were consistent with the scRNA‐seq results that IL‐36R was expressed in lung epithelial cells and endothelial cells but not in alveolar macrophages (Figure S2E, Supporting Information). Similarly, hIL‐36*γ* or mIL‐36*γ* significantly upregulated the mRNA and the protein levels of GCLM, GPXs and GSR in A549 cells, human airway organoids or HEK293‐mIL‐36R cells that were abolished by h/mIL‐36Ra, respectively (Figure 5C–E). Consistently, the GSH levels were increased by IL‐36*γ* which was abolished by IL‐36Ra in A549 cells, primary human organoid or mouse primary lung epithelial cells (Figure 5F), indicating that IL‐36*γ* and IL‐36Ra reciprocally regulate GSH biogenesis through modulating the expression of the related genes.

A primary function of GSH is to neutralize ROS and counteract oxidative stress.^[^ ^17^ ^]^ Cisplatin, glucose deprivation and H_2_O_2_ are potent inducers of oxidative stress that induce endogenous ROS or provide exogenous ROS.^[^ ^16^, ^45^ ^]^ Interestingly, we found that IL‐36*γ* significantly inhibited the upregulation of ROS in as well as cell death of A549 cells or HEK293‐mIL‐36R cells treated with cisplatin, glucose deprivation or H_2_O_2_ treatment and such an inhibitory effect was abolished by IL‐36Ra (Figure 5G,H and Figure S6C,D, Supporting Information). In contrast, IL‐36*γ* did not rescue cell death induced by CHX and TNF*α* which promoted apoptosis without upregulation of ROS (Figure S6E, Supporting Information). These data together suggest that IL‐36*γ* promotes GSH biogenesis and inhibits oxidative stress‐induced cell death which is counteracted by IL‐36Ra.

### 2.6 Inhibition of IL‐36*γ* Maturation Alleviates NSCLC Progression

**Figure 6:**
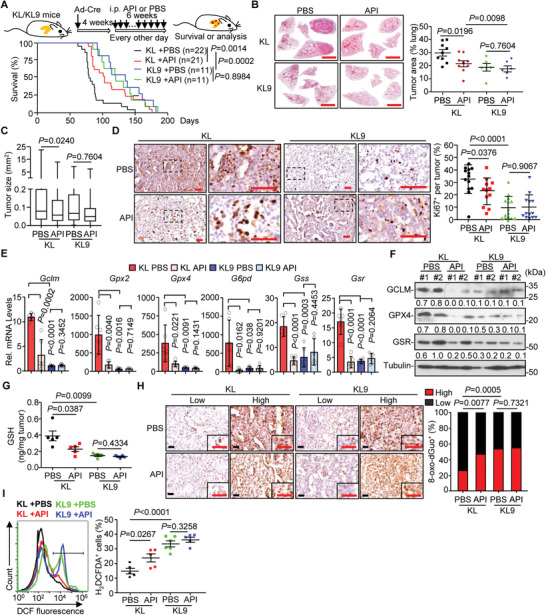
Inhibition of IL‐36*γ* maturation alleviates NSCLC progression. A) Schematic illustration of tumor induction and API treatment (upper) and the survival of mice (lower). KL or KL9 mice that were intranassally injected with Ad‐Cre for 4 weeks followed by intraperitoneal injection of PBS (200 µL, *n* = 22, and 11 mice for KL and KL9, respectively) or API (100 µg in 200 µL per mouse, *n* = 21 and 11 mice for KL and KL9, respectively) every other day for 6 weeks. B) Images (left) and quantification analysis (right) of HE staining of tumor‐burdened lungs of KL (*n* = 9 and 9 mice for PBS and API, respectively) and KL9 (*n* = 6 and 6 mice for PBS and API, respectively) mice treated as in (A). C) Box plot of individual tumor size in the tumor‐burdened lungs of KL (*n* = 9 and 9 mice for PBS and API, respectively) and KL9 (*n* = 6 and 6 mice for PBS and API, respectively) mice treated as in (A). D) Images (left) and quantification analysis (right) of Ki67 staining in individual lung tumors of KL (*n* = 13 and 12 mice for PBS and API, respectively) and KL9 (*n* = 15 and 13 mice for PBS and API, respectively) mice treated as in (A). E) qRT‐PCR analysis of the indicated genes or proteins in lung tumors from KL (*n* = 6 and 6 mice for PBS and API, respectively) and KL9 (*n* = 6 and 6 mice for PBS and API, respectively) treated as in (A). F) Immunoblot analysis of *Gclm*, *Gpx4*, and *Gsr* in lung tumors from KL (*n* = 2 and 2 mice for PBS and API, respectively) and KL9 (*n* = 2 and 2 mice for PBS and API, respectively) treated as in (A). G) GSH levels in lung tumors from KL (*n* = 5 and 5 mice for PBS and API, respectively) and KL9 (*n* = 4 and 4 mice for PBS and API, respectively) mice treated as in (A). H) Images (left) and quantification analysis (right) of 8‐oxo‐dGuo^+^ staining in lung tumors from KL (*n* = 9 and 9 mice for PBS and API, respectively) and KL9 (*n* = 6 and 6 mice for PBS and API, respectively) mice treated as in (A). I) Flow cytometry (left) and quantification analysis (right) of H_2_DCFDA staining of single‐cell suspensions of lung tumors from KL (*n* = 5 and 5 mice for PBS and API, respectively) and KL9 (*n* = 6 and 5 mice for PBS and API, respectively) mice treated as in (A). Graphs show mean ± SEM (B–E,G,I). Two‐tailed student's *t*‐test (B–E,G,I) or Log‐Rank analysis (A). Scale bars represent 5 mm (B) or 50 µm (D,H), respectively. Data are combined results of three independent experiments (A) or representative results of two independent experiments (B–I).

Elastase‐mediated cleavage at the N‐termini of IL‐36 cytokines is critical for the maturation and activation, which can be blocked by the z‐Ala‐Pro‐Ile (API) peptide.^[^ ^46^, ^47^ ^]^ Administration of API prolonged survival and inhibited lung tumor progression of KL mice that were intranasally injected with Ad‐Cre (**Figure** **6**A–C). In contrast, the survival and lung tumor progression of KL9 mice after tumor induction were not affected by API treatment (Figure 6A–C), indicating IL‐36*γ* as a primary target of API in the KL NSCLC model. In addition, API treatment significantly lowered the percentages of Ki67 staining and the levels of genes and proteins involved in GSH biogenesis in the lung tumors from KL mice but not KL9 mice after tumor induction (Figure 6D–F). Consistently, the GSH levels were reduced and the 8‐oxo‐dGuo levels and the DCF fluorescence were increased in API‐treated KL mice compared with the controls, whereas the GSH levels, 8‐oxo‐dGuo levels and the DCF fluorescence were comparable between the PBS‐ and API‐treated KL9 mice (Figure 6G–I). These data suggest that inhibition of IL‐36*γ* maturation effectively enhances the oxidative stress and alleviates NSCLC progression.

### 2.7 Neutralization of IL‐36 Inhibits NSCLC Progression

**Figure 7:**
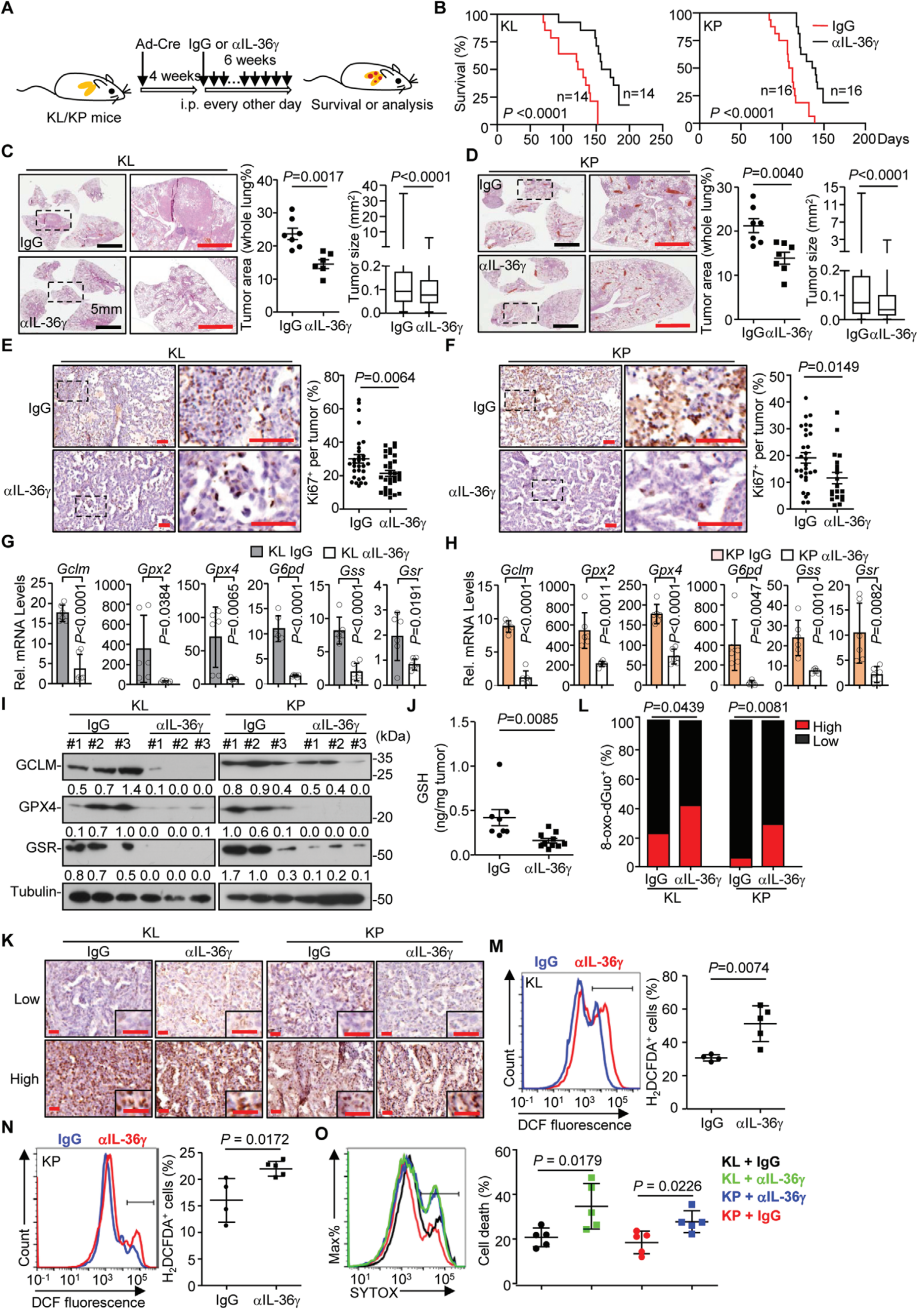
Neutralization of IL‐36*γ* inhibits NSCLC progression. A) Schematic illustration of tumor induction and *α*IL‐36*γ* treatment. KL/KP mice were intranasally injected with Ad‐Cre for 4 weeks followed by intraperitoneal injection of control IgG or *α*IL‐36*γ* (100 µg in 200 µL PBS per mouse) every other day for 6 weeks. B) Kaplan–Meier survival curves for KL (left, *n* = 14 and 14 mice for IgG or *α*IL‐36*γ*, respectively) or KP (left, *n* = 16 and 16 mice for IgG or *α*IL‐36*γ*, respectively) mice treated as in (A). C,D) Images of HE staining (left), tumor area (middle) and tumor size (right) of tumor‐burdened lungs of KL (C, *n* = 7 and 6 mice for IgG and *α*IL‐36*γ*, respectively) or KP (D, *n* = 7 and 7 mice for IgG and *α*IL‐36*γ*, respectively) mice treated as in (A). E,F) Images (left) and quantification analysis (right) of Ki67 staining in lung tumors from KL (E, *n* = 30 and 32 mice for IgG and *α*IL‐36*γ*, respectively) or KP (F, *n* = 29 and 20 mice for IgG and *α*IL‐36*γ*, respectively) mice treated as in (A). G,H) qRT‐PCR analysis of the indicated genes for GSH metabolism in lung tumors from KL (G, *n* = 6 and 6 mice for IgG and *α*IL‐36*γ*, respectively) or KP (H, *n* = 6 and 6 mice for IgG and *α*IL‐36*γ*, respectively) mice treated as in (A). I) Immunoblot analysis of GCLM, GPX4 and GSR in lung tumors from KL (left, *n* = 3 and 3 mice for IgG and *α*IL‐36*γ*, respectively) or KP (right, *n* = 3 and 3 mice for IgG and *α*IL‐36*γ*, respectively) mice treated as in (A). J) GSH levels in the lung tumors of KL (*n* = 8 and 10 mice for IgG or *α*IL‐36*γ*, respectively) mice treated as in (A). K,L) Images (K) and quantification analysis (L) of 8‐oxo‐dGuo staining in lung tumors of KL (*n* = 7 and 6 mice for IgG and *α*IL‐36*γ*, respectively) and KP (*n* = 7 and 7 mice for IgG and *α*IL‐36*γ*, respectively) mice treated as in (A). M–N) Flow cytometry (left) and quantification analysis (right) of H_2_DCFDA staining in single‐cell suspensions of lung tumors from KL (M, *n* = 4 and 5 mice for IgG and *α*IL‐36*γ*, respectively) or KP (N, *n* = 4 and 5 mice for IgG and *α*IL‐36*γ*, respectively) mice treated as in (A). O) Flow cytometry (left) and quantification analysis (right) of SYTOX Green staining in KP (*n* = 5 and 5 mice for IgG and *α*IL‐36*γ*, respectively) or KL (*n* = 5 and 5 mice for IgG and *α*IL‐36*γ*, respectively) treated as in (A). Graphs show mean ± SEM (C–H,J,L–O). Two‐tailed student's *t*‐test (C–H,J,L–O) or Log‐Rank analysis (B). Scale bars represent 5 mm (C,D, left), 2 mm (C,D, right), or 50 µm (E,F,K), respectively. Data are combined results of two independent experiments (B) or representative results of two independent experiments (C–O).

To directly and specifically block IL‐36*γ* in vivo, we generated a polyclonal antibody against IL‐36*γ* by immunizing rabbits followed by affinity purification. The antibody inhibited IL‐36*γ*‐ but not IL‐36*α*‐, IL‐36*β*‐, or TNF‐induced activation of NF‐*κ*B reporter in HEK392‐mIL‐36R cells (Figure S7A,B, Supporting Information), indicating high specificity and neutralizing activity of the generated antibody. Interestingly, intraperitoneal injection of anti‐IL‐36*γ* significantly prolonged the survival of KL or KP mice compared to the IgG‐treated mice (**Figure** **7**A,B). Consistently, the tumor development in the lungs of KL or KP mice and the Ki67^+^ staining in the lung tumors were substantially inhibited by anti‐IL‐36*γ* treatment (Figure 7C–F), indicating that neutralizing IL‐36*γ* inhibits NSCLC progression. Consistently with this notion, anti‐IL‐36*γ* treatment downregulated the expression of GSH biogenesis genes and proteins, and reduced the levels of GSH in the lung tumors from KL or KP mice at 10 weeks after tumor induction (Figure 7G–J). Conversely, the 8‐oxo‐dGuo levels and the DCF fluorescence in lung tumors from KL or KP mice were increased by anti‐IL‐36*γ* treatment compared to IgG treatment (Figure 7K–N). Consistently, the levels of SYTOX Green staining were increased by anti‐IL‐36*γ* treatment compared to IgG treatment (Figure 7O). Together, these data suggest that neutralization of IL‐36*γ* promotes oxidative stress in the lung tumors and thereby efficiently inhibits NSCLC development.

## 3 Discussion

IL‐36R signaling has been implicated in various inflammatory diseases such as psoriasis and colitis by modulating the production of pro‐inflammatory cytokines and chemokines.^[^ ^29^, ^30^, ^31^ ^]^ A phase I clinical trial with the IL‐36R antibody (Spesolimab) for generalized pustular psoriasis (GPP) has achieved promising outcomes and clinical trials for inflammatory bowel disease are on the way.^[^ ^48^ ^]^ In this study, we have discovered a pro‐inflammation‐independent role of IL‐36R signaling in NSCLC development (Figure S7C, Supporting Information). Specifically, IL‐36*γ*, an agonist for IL‐36R signaling, upregulated GSH levels by promoting expression of downstream genes involved in GSH biogenesis, which alleviated ROS and oxidative stress and potentiated cell survival and proliferation. Consequently, the progression of lung cancer in KL and KP NSCLC mouse models were accelerated and the survival time of tumor‐bearing KL and KP mice was shortened. Conversely, IL‐36Ra functioned oppositely in regard of GSH production, ROS resolution and NSCLC progression by antagonizing IL‐36R signaling. Therefore, blocking or antagonizing IL‐36R signaling may provide effective therapeutic interventions for mutated KRAS‐driven NSCLC. In this context, our preclinical data with anti‐IL‐36*γ* to neutralize IL‐36*γ* or elastase inhibitor to block IL‐36*γ* maturation have demonstrated that targeting IL‐36*γ* significantly alleviated NSCLC progression and prolonged the survival of KL or KP mice after tumor induction.

It has been shown that the expression of IL‐36*γ* is upregulated in inflamed skin and in colon tissues.^[^ ^32^, ^33^, ^34^, ^35^, ^36^, ^37^ ^]^ Here, we found that IL‐36*γ* was upregulated in NSCLC tumor tissues compared to the normal lung tissues and the levels of *Il1f9* were higher in advanced tumors than in early‐stage tumors from the KL mice. ChIP analysis suggested that the NF‐*κ*B subunit p65 and the AP‐1 subunit cJun bound to the promoter of *Il1f9* promoter in the tumor tissues but not in the normal lungs. In this context, various stimuli that activate NF‐*κ*B and AP‐1 have been implicated in upregulation of IL‐36*γ*, including pattern‐recognition receptors (PRRs)‐mediated signaling and pro‐inflammatory cytokines‐mediated signaling that can be generated by enhanced genome instability or cell death in accompany with tumor progression.^[^ ^29^, ^49^, ^50^ ^]^ Alternatively, ROS are increased in tumor cells and have been shown to activate NF‐*κ*B and AP‐1,^[^ ^17^ ^]^ which might upregulate IL‐36*γ* for GSH biogenesis and adaptation to oxidative stress (discussed below). A previous report has shown that ectopic expression of IL‐36*γ* in tumor cells promotes Th1 cell polarization, CD8^+^ T cell activation and anti‐tumor immunity.^[^ ^44^ ^]^ However, results from our scRNA‐seq analysis suggested that *Il1f9* was barely expressed in epithelial tumor cells in KL mouse model, which is consistent with recent studies.^[^ ^51^, ^52^ ^]^ In addition, *Il1rl2* was expressed in neutrophils, endothelial cells, and epithelial tumor cells but not in T cells. These observations indicate that neutrophils, endothelial cells, and epithelial tumor cells in the NSCLC tumor tissues are the major target cells of IL‐36*γ* and that the crosstalk among the IL‐36*γ*‐producing cells and IL‐36R‐expression cells critically regulates NSCLC progression. In this context, the percentages or numbers of IL‐17A‐ or IFN*γ*‐producing CD4^+^, CD8^+^, or *γδ*TCR^+^ cells in the lung tumors were comparable between KL and KL9 or KL and KL5 mice, indicating a dispensable role of IL‐36R signaling in T cell polarization, activation or expansion in the NSCLC mouse model.

It is acknowledged that IL‐36*γ* triggers and IL‐36Ra antagonizes IL‐36R signaling to modulate the production of pro‐inflammatory cytokines and chemokines.^[^ ^29^, ^30^, ^31^ ^]^ However, IL‐36*γ*‐ and IL‐36Ra‐deficient lung tumors from KL or KP mice exhibited similar down‐regulation patterns of pro‐inflammatory cytokines and chemokines compared to the controls. In contrast, the genes involved in GSH biogenesis and regeneration and the levels of GSH were downregulated and upregulated in IL‐36*γ*‐ and IL‐36Ra‐deficient lung tumors, respectively. In addition, IL‐36*γ* upregulated expression of these genes in cells and in vivo and such an effect was counteracted by IL‐36Ra. Consistently, the IL‐36*γ* staining was positively and negatively correlated with glutathione biosynthesis and ROS in human NSCLC tumor biopsies respectively, and *IL1F9* ^hi^ *IL1F5* ^low^ expression pattern predicted poor prognosis of NSCLC patients. In addition, the tumor progression was accelerated in KL and KL9 mice by the antioxidant NAC to a similar level of that in KL5 mice treated with saline, and NAC treatment did not affect NSCLC progression in KL5 mice, indicating that IL‐36*γ* and IL‐36Ra reciprocally regulate NSCLC progression by modulating the ROS levels and oxidative stress. IL‐36R signaling induces the activation of NF‐*κ*B and AP‐1, both of which play essential roles in the induction of genes whose products catalyze GSH biogenesis.^[^ ^53^ ^]^ Other transcription factors including NRF2, BACH1, FOXOs, PGC‐1*α*, and HIF‐1*α* are involved in GSH biogenesis, ROS resolution, and tumorigenesis.^[^ ^54^, ^55^, ^56^, ^57^, ^58^ ^]^ Whether and how these transcription factors are associated with IL‐36*γ*‐mediated upregulation of GSH biosynthesis or salvage requires further investigations.

Excessive ROS induces oxidative stress that leads to aberrant oxidation of lipids, proteins, and nucleic acids and promotes cell death.^[^ ^17^ ^]^ Consistently with the notion that IL‐36*γ* upregulated GSH levels to neutralize ROS which was antagonized by IL‐36Ra, the lung tumor cells from IL‐36*γ*‐ or IL‐36Ra‐deficient NSCLC mouse models were more or less prone to death than the controls, respectively. Chemotherapy and radiotherapy are common cancer treatment modalities which kill tumor cells by directly inducing DNA damage or cell cycle arrest or indirectly stimulating ROS.^[^ ^59^, ^60^, ^61^ ^]^ Recent studies have shown that immune checkpoint blockade (ICB) therapies promote tumor regression by facilitating T or NK cell‐mediated killing of tumor cells or inducing lipid ROS in tumor cells.^[^ ^62^, ^63^ ^]^ In addition, IFN*γ* released from CD8^+^ T cells promotes tumor cell lipid peroxidation and ferroptosis by downregulating the expression of glutamate‐cystine antiporter system X_c_^−^ to impair the uptake of cystine in tumor cells,^[^ ^63^ ^]^ indicating crucial roles of ROS in ICB therapy‐mediated tumor regression. However, the passive cell death and the upregulated ROS can activate NF‐*κ*B and AP‐1 that promote induction of IL‐36*γ* as a strategy for drug resistance. Therefore, combination of anti‐IL‐36*γ* with the traditional treatments may improve the efficacy of chemo‐, radio‐, or immune‐therapies and overcome the drug resistance for NSCLC patients. Taken together, these findings highlight potential therapeutic intervention for NSCLC by targeting IL‐36*γ* or blocking IL‐36R signaling.

## 4 Experimental Section

### Human NSCLC Samples

Three cohorts of human NSCLC samples were collected and analyzed in this study.^[^ ^43^ ^]^ Cohort 1 was collected from June to August of 2013 containing 10 paired NSCLC tumor and normal tissues that were used to screen differentially expressed cytokines. Cohort 2 was collected from November of 2013 to March of 2014 containing 43 paired NSCLC tumor and normal tissues that were used for confirmation of the screened cytokines from Cohort 1. These tumor and normal tissues (≈0.2 g) were washed with PBS, immersed in TRIzol and frozen in liquid nitrogen immediately after surgery at the Department of Thoracic Surgery, Tongji Hospital. Cohort 3 (containing 127 samples) was paraffin‐embedded tumor and normal tissues collected from September of 2013 through April of 2014 at the Department of Thoracic Surgery, Tongji Hospital, Tongji Medical College, Huazhong University of Science and Technology. The clinical information of patients from the three Cohorts was included or summarized in Tables S1, S2, S6, Supporting Information. All cases were re‐reviewed by pathologists from the Department of Pathology of Tongji Hospital for the confirmation of tumor histology and tumor content. This study was approved by the Institutional Review Committee of Tongji Hospital, Tongji Medical College, Huazhong University of Science and Technology, and the Medical Ethic Committee of the School of Medicine, Wuhan University.

### Mice

*Kras* ^LSL‐G12D/+^ (#0 08179), *Tp53* ^fl/fl^ (#0 08462), *Lkb1* ^fl/fl^ (#01 4143) mice were purchased from the Jackson Laboratory as previously described.^[^ ^43^ ^]^ C57BL/6 mice were purchased from GemPharmatech Co., Ltd (Nanjing, China). Il1f9^+/−^ (#032395‐UCD) and *Il1f5* ^+/−^ (#032393‐UCD) mice were obtained from the Mutant Mouse Resource and Research Center (MMRRC) and were crossed with C57BL/6 mice for six generations before subsequent studies. The *Il1f9^−/‐^* and *Il1f5^−/−^* mice on C57BL/6 mice background were crossed with *Kras* ^LSL‐G12D/+^ *Tp53* ^fl/fl^ (KP) and *Kras* ^LSL‐G12D/+^ *Lkb1* ^fl/fl^ (KL) to obtain *Kras* ^LSL‐G12D/+^ *Tp53* ^fl/fl^ *Il1f9^−/−^* (KP9) or *Kras* ^LSL‐G12D/+^ *Tp53* ^fl/fl^ *Il1f5^−/‐^* (KP5) and *Kras* ^LSL‐G12D/+^ *Lkb1* ^fl/fl^ *Il1f9^−/−^* (KL9) or *Kras* ^LSL‐G12D/+^ *Lkb1* ^fl/fl^ *Il1f5^−/‐^* (KL5) mice for maintenance and experiments. All mice were housed in the specific pathogen‐free animal facility at Wuhan University with a 12‐hour dark/12‐hour light cycle and fed with standard food and water. All animal experiments were performed in accordance with protocols approved by the Institutional Animal Care and Use Committee of Wuhan University.

### Tissue Microarray Preparation

Tissue microarray was prepared as previously described.^[^ ^43^, ^64^, ^65^ ^]^ In brief, tumor and normal tissues from NSCLC patients with wedge resection or pulmonary lobectomy were fixed in 4% paraformaldehyde and embedded into paraffin blocks. The paraffin blocks were punched out of the selected regions based on a hematoxylin‐eosin (HE) staining analysis. The punched samples were 1.5 mm in diameter and 4–6 mm in length and assembled into a new paraffin block. The tissue microarrays were sectioned (4 µm) and stained with H&E to confirm the histological results.

### Induction of Tumor of KP or KL Mouse Models

The experiments were performed as previously described.^[^ ^43^ ^]^ Eight‐to‐ten‐week‐old KP, KP9 or KP5, KL, KL9, or KL5 mice were anesthetized by intraperitoneal injection of 1% sodium pentobarbital (w/v = 1:7), followed by intranasal injection of Ad‐Cre viruses (Obio Technology, Shanghai) (1–2 × 10^6^ pfu in 60 µL PBS per mouse). The survival of mice was recorded until the end of the study. Alternatively, at the indicated time points after infection, mice were euthanized and the tumor bearing lungs or dLNs were removed for subsequent analysis.

### Hematoxylin and Eosin Staining and Immunohistochemistry

Hematoxylin and eosin (HE) staining and immunohistochemistry (IHC) staining was performed as previously.^[^ ^43^ ^]^ Briefly, mouse lung tissues were fixed with 1 mL 4% paraformaldehyde (PFA), fixed for 4 h and dehydrated in diluted ethanol for 1 h for each gradient. The lungs were embedded in paraffin and sectioned (5 µm) for subsequent staining with hematoxylin and eosin (Beyotime Biotech). Images were acquired using a Aperio VERSA 8 (Leica) multifunctional scanner. Tumor burden and individual tumor size were determined through ImageScope (Leica) as described previously.^[^ ^43^ ^]^

For IHC staining, slides were deparaffinized in xylene, and rehydrated in 100%, 95%, and 75% ethanol for 5 min. Antigen retrieval was performed by heating slides in a microwave for 30 min in sodium citrate buffer (pH 6.0) or 0.5 mm EDTA buffer (pH 8.0). The sections were cooled down naturally to room temperature and quenched in 3% hydrogen peroxide to block endogenous peroxidase activity. The primary antibody diluted in PBS containing 1% BSA was incubated at 4 °C overnight followed by Maixin_Bio Detection Kit peroxidase/diaminobenzidine (DAB) rabbit/mouse (Kit‐9710, DAB‐0031; Maixi_Bio, Fuzhou) according to the manufacturer's instructions. Subsequently, sections were counterstained with hematoxylin (Beyotime Biotech) for 1 min and coverslipped. The information and dilution of antibodies were used has been listed in Table S8, Supporting Information. Images were acquired with the Aperio VERSA 8 (Leica) multifunctional scanner. The intensities of DAB staining were measured and quantified with integrated optical density or cell intensity by Image Pro Plus 6 (Media Cybernetics) as described.^[^ ^43^ ^]^

For 8‐oxo‐dGuo positivity analysis, the low or high 8‐oxo‐dGuo staining image showed one represent field of different tumors (1–5 mm^2^) rather than different regions within one tumor. The intensities of 8‐oxo‐dGuo staining of the randomly selected field (≈0.2 mm^2^, shown in the figures) in the tumor were quantified by Image J Plus 6 software. The obtained value was divided by the cell numbers in the field which was recorded as the integral optical density (IOD) of the specific field. The 8‐oxo‐dGuo positivity of each tumor was calculated by averaging the IOD values of five different fields within the tumor. The tumors with 8‐oxo‐dGuo positivity >200 were classified as high 8‐oxo‐dGuo staining and those <200 were classified as low 8‐oxo‐dGuo staining. The percentage of low or high 8‐oxo‐dGuo staining of each mouse was calculated by the formula: number [8‐oxo‐dGuo positivity <200] or number [8‐oxo‐dGuo positivity >200]/(number[8‐oxo‐dGuo positivity <200] + number [8‐oxo‐dGuo positivity >200]). The graphs showed the average percentages of high or low 8‐oxo‐dGuo positivity of multiple experimental mice and the statistical analyses were performed with two‐tailed student's *t*‐test.

### Quantitative Real‐Time PCR

These experiments were performed as previously described.^[^ ^43^, ^66^, ^67^ ^]^ Total RNA was extracted from tumor or normal tissues or cells using TRIzol reagent (Invitrogen), and the first‐strand cDNA was reversed‐transcribed with All‐in‐One cDNA Synthesis SuperMix (Biotool). Gene expression was examined with a Bio‐Rad CFX Connect system by a fast two‐step amplification program with 2× SYBR Green Fast qPCR Master Mix (Biotool). The Ct values of each gene (Ct_gene_) and *β*‐actin (Ct_actin_) were recorded by the Bio‐Rad CFX Connect system and subsequently calculated by averaging the three technical replicates. The relative expression levels were calculated by the following formula: 2^(Ctactin‐Ctgene)^ × 10^3^. Each dot in the graphs represented data of multiple mixed tumors from one mouse. Gene‐specific primers are listed in Table S9, Supporting Information.

### Immunoblot Assays

The immunoblot assays were performed as described.^[^ ^64^, ^66^, ^67^ ^]^ Briefly, whole cell lysates or tumor tissues were prepared in lysis buffer supplemented with protease and phosphatase inhibitors (Roche). The lysates were centrifuged at 12 000 × *g* for 10 min at 4 °C followed by quantification with the BCA Protein Assay Kit (Thermo Fisher Scientific, 23 225). Equal amounts of protein were subject to SDS‐PAGE followed by transfer and immunoblot. Primary antibodies were anti‐GCLM (ab126704, Abcam), anti‐GSR (A12070, ABclonal), anti‐GPX4 (ab125066, Abcam), anti‐G6PD (HPA000834.Sigma), anti‐Tubulin (KM9003, Sungene Biotech), and secondary antibodies were HRP‐conjugated goat‐anti‐mouse or rabbit IgG (Thermo Fisher Scientific, PA1‐86717, and SA1‐9510, respectively).

### Preparation of Single Cell Suspensions from Tumor‐Burdened Lungs

Tumor‐burdened lungs from KL, KL5, or KL9 mice were perfused through alveolar lavage and cardiac lavage with PBS. The tumor‐burdened lungs or lung tumors were isolated and cut into small pieces (1–2 mm in diameter) and transferred into a gentleMACS C Tube with the enzyme mix containing 2.35 mL of DMEM, 100 µL of Enzyme D, 50 µL of Enzyme R, and 12.5 µL of Enzyme A from a Tumor Dissociation Kit (Miltenyi Biotech, Cat# 130‐096‐730). The C Tube was tightly closed and attached onto the sleeve of the gentleMACS Octo Dissociator (Miltenyi Biotech) with the tumor isolation program. After termination of the program, C tube was detached from the Dissociator and incubated at 37 °C with continuous rotation at 220 rpm for 40 min. Then repeat the tumor isolation program twice and perform a short spin up to 1500 g to collect the sample at the bottom of the tube. After dissociation, the sample re‐suspended was applied to MACS SmartStrainers (70 µm) to prepare single‐cell suspension.

### Chromatin Immunoprecipitation (ChIP) Assays

These experiments were performed as previously described.^[^ ^43^, ^68^ ^]^ The single cell suspensions of lung tumors were fixed with 1% formaldehyde and quenched by glycine and then washed three times with PBS and then harvested in ChIP lysis buffer (50 mm Tris·HCl pH 8.0, 1% SDS, 5 mM EDTA) followed by sonication to generate DNA fragments of 300–500 bp. The lysate was centrifuged at 12 000 rpm for 15 min at 4 °C and were diluted with ChIP dilution buffer (20 mm Tris·HCl, pH 8.0, 150 mm NaCl, 2 mm EDTA, 1% Triton X‐100) (4:1 volume). The resulting lysate was then incubated with Protein G agarose and anti‐p65 (SC‐372, Santa Cruz Biotechnology), anti‐cJun (9165S, Cell Signaling Technology), anti‐STAT3 (SC‐8019, Santa Cruz Biotechnology), anti‐pSTAT3 (9145S,Cell Signaling Technology) or control IgG at 4 °C for 4 h. DNA was eluted using ChIP elution buffer (0.1 m NaHCO_3_, 1% SDS, 30 µg mL^−1^ proteinase K) by incubation at 65°C overnight, and the DNA was purified with a DNA purification kit (TIANGEN). The purified DNA was assayed by quantitative PCR using the SFX connect system with the 2× SYBR Green fast qPCR master mix kit (Biotool). The qPCR primer sequences of *Il1f9* promoter were listed in Table S8, Supporting Information.

### Preparation of Lung‐Infiltrated Lymphocytes

The obtained single‐cell suspensions of tumor‐burdened lungs were centrifuged at 1500 × *g* for 5 min at room temperature, and the precipitants were re‐suspended with 40% Percoll (Cat#17‐0891‐09, GE Healthcare) in PBS (v/v). The suspension was centrifuged at 1500 × *g* for 20 min at room temperature and the supernatant was discarded. The precipitants containing LILs were re‐suspended in 10% FBS DMEM containing PMA (50 ng mL^−1^, P8139, Sigma), Ionomycin (500 ng mL^−1^, I0634, Sigma), Golgi‐stop (1:1000, Cat# 554 724, BD Biosciences) and cultured for 4 h at 37 °C, 5% CO_2_, followed by staining and flow cytometry analysis.

### Flow Cytometry Analysis

Flow cytometry protocol has been previously described.^[^ ^43^ ^]^ The single‐cell suspensions of tumor‐burdened lungs, bronchial dLN or the obtained LILs were re‐suspended in FACS buffer (PBS, 1% BSA) and blocked with anti‐mouse CD16/32 antibodies for 10 min prior to staining with the antibodies of the surface markers. For intracellular cytokine staining, cells were fixed and permealized with a fixation and permeabilization solution kit (Cat# 424 401, Biolegend) followed by staining with the specific antibodies against intracellular cytokines. Antibodies used for flow cytometry analysis were listed in Table S9, SUpporting Information. Flow cytometry data were acquired on a FACSCelesta or LSRFortessaX20 flow cytometer (BD Biosciences) and analyzed with FlowJo X software (TreeStar).

### Measurement of GSH and GSSG Levels

Frozen tumor tissues (≈50 mg) were homogenized in ultrapure water with Zirconium Oxide Beads (Next Advance, Inc.) by TissueLyser48 (Shanghai Jingxin Industrial Development Co.), then mixed with acetonitrile. The denatured proteins were separated by centrifuging at 12 000 × *g* for 10 min. The level of GSH in the supernatants was identified by Thermo Scientific Q Exactive Plus hybrid quadrupole‐Orbitrap coupled to Thermo Scientific UltiMate 3000 HPLC system. Meanwhile, the standard curve was prepared by quantification of the serially diluted standard GSH or GSSG (Sigma‐Aldrich) assayed under the same conditions. Specifically, the lysates or standard samples were separated on Hypercarb Porous Graphitic Carbon HPLC Columns (2.1 mm inner diameter, 100 mm length, 3 µm particles, Thermo Scientific) in a 10 min gradient from 5% to 95% in acetonitrile. Both columns were at 35 °C and 5 µL of each sample was injected into the LC‐MS with a flow rate of 0.2 mL min^−1^.

Mass spectra were acquired with parallel reaction monitoring (PRM) and full MS scan. For PRM, the key parameters were as following: isolation window, 4.0 m/z, resolution, 35 000, maximum injection time, 100 ms, and AGC target value, 2E5. For Full MS, the key parameters were as following: resolution, 70 000, maximum injection time, 200ms, AGC target value, 3E6, and scan range, 200–800 m/z. The data analysis was performed with the Thermo Scientific Xcalibur software (version 4.3).

### Analysis of Reactive Oxygen Species Levels and Cell Viability

Single cell suspensions of lung tumors were suspended in 3 mL Red Blood Cell Lysis Buffer (Sigma‐Aldrich) for 1 min, and diluted with equal volume of PBS to end the reaction. The cells were centrifuged, washed and stained with 100 µm H_2_DCFDA (Molecular Probes, Invitrogen) or 30 nm SYTOX Green Dead Cell Stain (Thermo Fisher Scientific), and incubated for 15 min at 37 °C in a tissue culture incubator. Cells were centrifuged, washed and resuspended in 200 µL fresh PBS followed by flow cytometry analysis.

To quantify the cytosolic ROS levels and cell viability in cultured cells, the cells with various treatment were harvested and stained with 100 µm H_2_DCFDA (in 200 µl PBS) or 30 nm SYTOX Green Dead Cell Stain for 15 min at 37 °C in a tissue culture incubator. Cells were washed and resuspended in 200 µL fresh PBS and followed with flow cytometry analysis. Flow cytometry data were acquired on a FACSCelesta flow cytometer (BD Biosciences, BD FACSDiVa Software v8.0.1.1) and analyzed with Flowjo 10.6.2 software (TreeStar).

### IL‐36*γ* and IL‐36Ra Treatment In Vivo

To verify IL36*γ*‐induced pulmonary response in vivo, 8‐week‐old male C57BL/6 mice were anaesthetized with isoflurane followed by intranasal injection of IL‐36*γ* (0.5 µg in 50 µL PBS per mouse, 6996‐IL‐010, R&D system), or IL‐36*γ* plus IL‐36Ra (210‐36RA, Peprotech, 0.5 µg for each per mouse in 50 µL PBS) or PBS (50 µL). 24 h later, the mice were sacrificed and the whole lungs were perfused through alveolar lavage and cardiac lavage with PBS. The lung tissues were collected in ice‐cold TriZol immediately for subsequent qRT‐PCR or immunoblot analysis.

### Isolation of Mouse Lung Epithelial Cells and Alveolar Macrophage

Mouse primary lung epithelial cells and alveolar macrophages were isolated as described previously.^[^ ^43^ ^]^ Lungs from 8‐week‐old female C57B/6 mice were perfused through cardiac lavage with PBS. For mouse lung epithelial cells, dispase solution (2 mL at 3.6 unit mL^−1^; 17105–41; Gibco) was instilled into the lungs through a tracheal catheter. Lungs were removed from mice and incubated in the dispase solution for 1 h at room temperature. The lungs were microdissected and cell suspensions were filtered through nylon monofilament. The recovered cells were centrifuged at 1500 × *g* for 5min and resusbended in PBS containing 1.5% FBS. The cells were incubated with anti‐CD45 microbeads for 30 min at 4 °C and the CD45^+^ cells were depleted by flow‐through a magnet column (Miltenyi Biotec). The resulted cells were resuspended in DMEM containing 10% FBS, and 1% streptomycin‐penicillin and were seeded into 12‐well plates at a density of 5 × 10^5^ cells per well for overnight culture, followed by various treatments.

For mouse alveolar macrophages, the lungs were digested by collagen IV in vitro to prepare single‐cell suspension followed by staining with antibodies against CD11b, CD11c, H‐2K^b^, and CD103 and flow cytometry analysis. The CD11b^low^CD11c^high^H‐2K^b −^CD103^−^ cell population was collected as alveolar macrophage and stained with Typab blue to examine the viability. The resulted cells were resuspended in DMEM medium and seeded into 6‐well plates at a density of 5 × 10^6^ cells per well for overnight culture followed by various treatments.

### Cell Culture

HEK293 and A549 cells were cultured in DMEM containing 10% FBS and 1% streptomycin and penicillin for various experiments.

For glucose deprivation assays, cells were first cultured in 12‐well plate in glucose‐free DMEM (Life Technologies) supplemented with 25 mm d‐glucose (Sigma). 12 h later, the supernatants were removed and the cells were washed with pre‐warmed PBS and cultured in glucose‐free DMEM (1mL per well) for another 16 h followed by adding IL‐36*γ* (20 ng per well, 6996‐IL‐010 and 6835‐IL‐010 for mouse and human IL‐36*γ* respectively, R&D system) with or without IL‐36Ra (20 ng per well, 210–36RA, Peprotech for mouse, and 1275‐IL‐025, R&D system for human, respectively) or NAC (5 mm, HY‐B0215, MedChemExpress) for 8 h. The cells were subject to imaging or harvested for staining.

For cisplatin‐induced ROS accumulation assays, cells were cultured in 12‐well plate for 12 h followed by supplementation of 20 µm cisplatin (Cat #T1564, TOPSCIENCE E7781‐1MG) for 10 h. The cells were then treated with IL‐36*γ* with or without IL‐36Ra (20 ng per well) or with NAC (5 mm) for 8 h. The cells were subject to imaging or harvested for staining.

For H_2_O_2_‐induced cell death, the cells were cultured in 12‐well plate in DMEM containing 10% FBS and 1% streptomycin and penicillin. 12 h later, the medium were supplemented with IL‐36*γ* with or without IL‐36Ra or with NAC for 7 h. Consequently, H_2_O_2_ (8 m, 2 µL per well) were added into the medium for 1 h. The cells were subject to imaging or harvested for staining.

For CHX and TNF*α*‐induced apoptosis, the cells were cultured in 12‐well plate in DMEM containing 10% fetal bovine serum and 1% streptomycin and penicillin for 12 h. The medium were supplemented with CHX (100 mg mL^−1^, 1:3000 in use, Enzo Biochem, ALX‐380‐269) and TNF*α* (10 ng per well, SRP2102‐10UG, Sigma‐Aldrich) with IL‐36*γ* or Z‐VAD‐FMK (50 µm, HY‐16658B, MedChemExpress). Ten hours later, the cells were subject to imaging or harvested for staining.

Phase contrast images were acquired using an OLYMPUS microscope equipped with a 10× phase‐contrast objective. Three independent fields were acquired for each experimental condition. Representative samples from one field of view were shown.

### Generation of Human Airway Organoids

The normal lung tissues (0.5−1 cm in diameter) were obtained from NSCLC patients who underwent surgery. The tissues were washed with wash buffer (Advanced DMEM/F12 containing 1× Glutamax, 10 mm HEPES, and antibiotics) (10 mL) and cut into small pieces followed by digestion with 2 mg mL^−1^ collagenase (Sigma Aldrich, C9407) in wash buffer on an orbital shaker at 37 °C for 1–2 h. The digested tissue suspension was sequentially sheared with 10‐mL Pasteur pipettes and filtered through the 100‐µm cell strainer (FALCON) and this step was repeated two times with 10 mL wash buffer containing 2% FBS. The flow‐through was centrifuged at 400 × *g* at 4 °C for 5 min. The erythrocytes in the pellet were lysed in 1 mL red blood lysis buffer for 2 min at room temperature before addition of 10 mL wash buffer containing 2% FBS and centrifugation at 400 × *g* at 4 °C for 5 min. The pellet was resuspended and embedded in matrigel (Growth Factor Reduced Basement Membrane Matrix; Corning, Cat# 354 234) and were seeded in 24‐well suspension culture plates (Greiner Bio‐One). After solidification, Matrigel droplets were maintained with culture medium (Table S10, Supporting Information) at 37 °C in a humidified incubator with 5% CO_2_. Medium was changed every 4 days and organoids were passaged every 1–2 weeks at a ratio 1:2–1:4. For passage, the generated organoids were resuspended in 2 mL culture medium and mechanically sheared by 1 mL pipettes followed by centrifugation at 400 × *g* at 4 °C for 5 min. The pellet was resuspended in 10 mg mL^−1^ growth factor reduced BME and reseeded as above described. Informed consents of the NSCLC patients were obtained and this study was approved by the Institutional Review Committee of Tongji Hospital, Tongji Medical College, Huazhong University of Science and Technology, and the Medical Ethic Committee of the School of Medicine, Wuhan University.

### Measurement of NADP^+^ and NADPH Levels

The levels of NADP^+^ and NADPH in lung tumors were determined by the commercially available kit (Sigma, #MAK312). Briefly, frozen tumor tissues (≈40 mg) of each sample were homogenized with either 200 µL NADP^+^ extraction buffer or NADPH extraction buffer for NADP^+^ or NADPH determination, respectively. The extracts were heated at 60 °C for 5 min and mixed with 40 µL Assay Buffer and 200 µL opposite extraction buffer followed by vortex. The extracts were centrifuged at 14 000 × *g* for 5 min. The supernatants were saved for quantification of NADP^+^ or NADPH. Meanwhile, the standard curves of NADP^+^ or NADPH were generated by serial dilution of the standard NADP^+^ (note: the standard curves for NADP+ and NADPH are identical, since NADPH in solution is unstable). For quantification of NADP^+^ or NADPH, the supernatants and the diluted standards (50 µL) were transferred into wells of a black flat‐bottom 96‐well plate and quickly mixed with 50 µL Working Reagent (consisting of 40 µL Assay Buffer, 1 µL of Enzyme A, 1 µL of Enzyme B, 10 µL of Glucose, and 5 µL of Probe). The fluorescence at *λ*_ex_ = 530 nm/*λ*_em_ = 585 nm was measured after 0 min “zero” (*F*_0_) and after a 30 min (*F*_30_) incubation at room temperature. The Δ*F* values of each standard or sample were calculated by subtracting *F*_0_ and *F*_30_. The NADP(H) concentration of the sample is computed as following: [NADP(H),(µm)] = (Δ*F*_sample_ – Δ*F*_blank_)/slope × *n*. Δ*F*_sample_ and Δ*F*_blank_ are the change in fluorescence intensity values of the sample and Blank, respectively. Slope is the slope of the standard curve and n is the diluted factor (if necessary).

### Construct Generation, Expression, and Purification of Mouse IL‐36*γ*

Mouse IL‐36*γ* (aa12‐191) was cloned into a pET‐30c expression vector containing an N‐terminal hexahistidine tag followed by a tobacco etch virus (TEV) protease recognition site. The construct was transformed into *Escherichia coli* (Rosetta 2). When the OD_600_ value of the cell cultures was ≈0.6–0.8, 0.1 mm isopropyl *β*‐d‐thiogalactoside (IPTG, 1122GR100, BioFroxx) was added into the medium for 18 h at 18 °C. The cells were lysed with high pressure crush, and the His‐TEV‐IL‐36*γ* protein was purified by Ni2^+^‐NTA chromatography. The hexahistidine tag was removed by digestion with 6xHis‐TEV (kindly provided by Dr. Lei Yin, Wuhan University), both of which were removed by a second Ni2^+^‐NTA chromatography step. Finally, the proteins were purified by gel filtration on a Superdex 200 column either in 50 mm imidazole (Cat: 8 142 230 250, Sigma) and 50 mm NaCl for crystallography or in 10 mm HEPES (pH 7.4), 150 mm NaCl, and 2 mm DTT for binding and functional assays.

### Immunization of IL‐36*γ* and Antibody Purification

The New Zealand rabbits were subcutaneously immunized with IL‐36*γ* (400 µg) and competent Freud's adjuvant at week 0. At week 1, 3 and 6, the rabbits were subcutaneously immunized with IL‐36*γ* (200 µg) and incompetent Freud's adjuvant. At week 0, 4, and 7, ≈2 mL blood was collected for ELISA analysis. At week 9, the rabbits were intravenously injected with IL‐36*γ* (400 µg). At week 10, the rabbits were euthanized and the blood was obtained to isolate the antisera by centrifuge of 1000 × *g* for 5 min at 4 °C. The antisera were cleared by further centrifuge at 20 000 × *g* for 20 min at 4 °C and diluted by ten times volume of the binding buffer (0.1 m phosphate buffer, 0.15 m NaCl, pH 8.0). The diluted antisera were loaded to the pre‐equilibrated Protein A column followed by washing with the binding buffer for three times. The anti‐IL‐36*γ* IgG were eluted with the elution buffer (0.2 m sodium citrate, pH 3.0) and dialyzed with PBS. The neutralization activity of *α*IL‐36*γ* was determined with NF‐*κ*B luciferase reporter assay. The control rabbit IgG were purchased from Yorogen (Wuhan) Biotech Ltd.

### Treatment with API, *α*IL‐36*γ* or NAC in Mouse Models

The z‐API peptide (Ala‐Pro‐Ile) was synthesized by GL Biochem (Shanghai) Ltd. For API treatment, 8‐week‐old KL and KL9 mice were infected intranasally with Ad‐Cre (2 × 10^6^ pfu in 60 µL PBS per mouse). At the fourth week after tumor induction, the mice were either intraperitoneally injected with PBS (Gibico, 200 µL) or API (100 µg in 200 µL PBS) every other day for 6 weeks followed by histological analysis, flow cytometry assays or survival observation.

For anti‐IL‐36*γ* antibody treatment, the KL and KP mice were intranasally infected with Ad‐Cre (2 × 10^6^ pfu in 60 µL PBS per mouse). Four weeks later, these mice were either intraperitoneally injected with control IgG or *α*IL‐36*γ* (100 µg in 200 µL PBS) every other day for 6 weeks followed by histological analysis, flow cytometry assays or survival observation.

For NAC (Acetyl‐cysteine) treatment, the KL, KL5, and KL9 mice were intranasally infected with Ad‐Cre (2 × 10^6^ pfu in 60 µL PBS per mouse). Four weeks later, the mice were either intraperitoneally injected with control saline (200 µL) or NAC (5 mg in 200 µL Saline, HY‐B0215, MedChemExpress) every other for 6 weeks followed by histological analysis or survival observation.

### mRNA‐Seq Analysis

Tumor‐burdened lungs from KL/KL5/KL9 mice and KP/KP5/KP9 mice were perfused through alveolar lavage and cardiac lavage with PBS. The lung tumors were collected and immediately homogenized in 2 mL of TRIzol (Invitrogen). Total RNAs were prepared and the quality of RNAs was determined by agarose gel electrophoresis and spectrophotometer analysis. Poly(A) mRNA was subsequently purified from 10µg total RNA using NEBNext Oligo d(T)_25_ Magnetic Beads Isolation Module. First‐strand complementary DNA was synthesized with NEBNext RNA First‐Strand Synthesis Module. NEBNext Ultra II Non‐Directional RNA Second Strand Synthesis Module was used for the synthesis of the complementary strand of first‐strand cDNA. The resulting double‐stranded DNA was purified and Vazyme TruePrep DNA Library Prep kit V2 was used to prepare libraries followed by sequencing on an Illumina Hiseq X Ten platform with 150‐bp paired‐end reads strategy (Novogene). Quality control of mRNA‐seq data was performed by using Fatsqc (v0.11.9) and low‐quality bases were trimmed by Trim_galore (0.6.4_dev). All RNA‐seq data were mapped to the mouse genome (Mus_musculus_Ensemble_94) by Hisat2 (v.2.0.5) and allowed a maximum of two mismatches per read. Gene expression level was calculated by FeatureCounts (v.2.0.0) with default parameters and normalized by FPKM (Fragments per Kilobase of exon model per Million mapped fragments). KEGG pathway analyses were performed with the differentially expressed genes of statistical significance.

To analyze a positive or negative enrichment of the indicated pathways, gene‐set enrichment analyses (GSEA) with the mRNA‐seq data was performed as previously described.^[^ ^64^ ^]^ In such analyses, the gene sets annotated in KEGG pathways (Table S5, Supporting Information) and their corresponding expression values (Table S4, Supporting Information) were analyzed through the GSEA software (http://www.gsea‐msigdb.org/gsea/index.jsp).

### Public Database Analysis

The original TCGA LUAD and LUSC RNA‐seq data and clinical data were downloaded from UCSC Xena (http://xena.ucsc.edu/) to examine the expression levels of *IL1F9* in LUAD (57 samples) or LUAD (49 samples) tissues and the paired non‐cancerous adjacent tissues (NATs) of NSCLC patients. These data were processed in R (version 4.0.1) with its basic functions. The related information was included in Table S3, Supporting Information. The microarray data of tumor tissues from LUAD (33 patients, TCGA.LUAD.sampleMap/AgilentG4502A_07_3) and LUSC patients (155 patients, TCGA.LUSC.sampleMap/AgilentG4502A_07_3) who were followed up for more than 10 years were downloaded to evaluate the correlation between levels of *IL1F9* or *IL1F5* and overall survival (183 of 188 patients had survival data). The expression levels of *IL1F9* or *IL1F5* above or below the median values were considered high or low expression, respectively. The Kaplan–Meier survival curves were generated with the GraphPad Prism 8.3.0 software. The related information was included in Table S7, Supporting Information.

### Single‐Cell mRNA‐seq

Single‐cell suspensions were prepared from lung tumors of KL mice after 10 weeks of tumor induction. Single‐cell RNA‐seq libraries were prepared with Chromium Single cell 3’ Reagent v3.0 Kits according to the manufacturer's protocol and loaded on the Chromium Single Cell Controller Instrument (10xGenomics) to generate single cell gel beads in emulsions (GEMs). About 10^5^ single cells were suspended in calcium‐ and magnesium‐free PBS containing 0.04% weight/volume BSA. About 15 000–20 000 cells were added to each channel with a targeted cell recovery estimate of 5000 cells (4400 and 2780 for #1 and #2 KL mice, respectively). After generation of GEMs, reverse transcription reactions were engaged barcoded full‐length cDNA followed by the disruption of emulsions using the recovery agent and cDNA clean up with DynaBeads Myone Silane Beads (Thermo Fisher Scientific). The cDNAs were then amplified by PCR with appropriate cycles which depend on the recovery cells. Subsequently, the amplified cDNA was fragmented, end‐repaired, A‐tailed, index adaptor ligated and library amplification. Then these libraries were sequenced on the Illumina sequencing platform (HiSeq X Ten), and 151 bp paired‐end reads were generated.

### Single‐Cell RNA‐seq Data Preprocessing

The Cell Ranger software pipeline (version 3.1.0) provided by 10xGenomics was used to demultiplex cellular barcodes, map reads to the genome and transcriptome using the STAR aligner, and downsample reads as required to generate normalized aggregate data across samples, producing a matrix of gene counts versus cells. The unique molecular identifier (UMI) count matrix is processed using the R package Seurat (version 3.2.0) with default parameters. After quality‐control,removing low‐quality cells and likely multiplet captures, which is a major concern in microdroplet‐based experiments, a criteria to filter out cells with UMI/gene numbers out of the limit of mean value ± 2 fold of standard devitation, which assuming a Guassian distribution of each cells’ UMI/gene numbers is further applied. Following visual inspection of the distribution of cells by the fraction of mitochondrial genes and ribosomal genes expressed, low‐quality cells where > 10% of the counts belonged to mitochondrial genes were further discarded. After applying these quality control criteria, 31 053 genes for the two mice remained and were included in subsequent analyses. Library size normalization was performed in Seurat on the filtered matrix to obtain the normalized count.

Top variable genes across single cells were identified using the method described previously.^[^ ^51^, ^52^ ^]^ Briefly, the average expression and dispersion were calculated for each gene, genes were subsequently placed into 11 bins based on expression. Principal component analysis was performed to reduce the dimensionality on the log transformed gene‐barcode matrices of top variable genes. Cells were clustered based on a graph‐based clustering approach, and were visualized in 2D using tSNE. Likelihood ratio test that simultaneously tested for changes in mean expression and in the percentage of expressed cells was used to identify differentially expressed genes between clusters.

### Transfection and Reporter Gene Assays

HEK293 cells were transiently transfected with NF‐*κ*B‐driven firefly luciferase reporter (100 ng), TK‐Renilla luciferase reporter (20 ng) and mIL‐36R (100 ng) using standard calcium phosphate precipitation. 20 h after transfection, the cells were stimulated with IL‐36*α*, IL‐36*β*, or IL‐36*γ* for 8 h followed by luciferase assays with a dual‐specific luciferase reporter kit (Promega). The activity of firefly luciferase was normalized by that of Renilla luciferase to obtain relative luciferase activity.

### Statistical Analysis

Graphs show mean ± SEM of different mice unless indicated otherwise. The numbers of sample sizes (*n*) represent mice, tumors or technical replicates in the related experiments unless indicated otherwise. IHC, quantification of GSH, GSSG, or NADP^+^/NADPH, and qRT‐PCR experiments were analyzed by two‐tailed Student's *t*‐test. For animal and human survival analysis, the Kaplan–Meier method was adopted to generate graphs and survival curves were analyzed by log‐rank analysis. RNA‐seq data were analyzed by DESeq2 and DAVID 6.8. For correlation analysis, Pearson correlation calculations were performed with Prism 8.3.0. Hypergeometric test was performed for KEGG pathway analysis. GSEA was used to rank the probes and analyze the enrichment based on t statistics (http://www.broadinstitute.org/gsea/). The heatmap of the signature genes was generated in GraphPad Prism 8.3.0 software based on their expression levels. All analyses above were performed using GraphPad Prism 8.3.0 software unless indicated otherwise. A *p* value (or an adjusted *p* value for multiple comparisons) <0.05 was considered statistically significant.

## Conflict of Interest

Ms. Zou Wang is an employee of Wuhan Biobank Co., Ltd and Mr. Yongbo Cheng is an employee of Elem Biotech Co., Ltd, Wuhan, China.

## Author Contributions

P.W. and W.Y. contributed equally to this work. B.Z. and D.L. designed and supervised the study; P.W. designed and performed the major experiments; P.W. and W.Y. performed core animal experiments; P.W. performed bioinformatics analyses and mechanisms studies; H.G. helped with flow cytometry, immunoblot, and cell culture; D.P. and Y.G. helped with mouse breeding and genotyping; H.G., Z.W., and Y.C. performed LC‐MS experiments; Y.D. collected human NSCLC samples; X.Y. and S.X. generated the human airway organoids; B.Z., P.W., and D.L. wrote the paper; all the authors analyzed data.

## Supporting information

Supporting Information

Supplemental Table 1

Supplemental Table 2

Supplemental Table 3

Supplemental Table 4

Supplemental Table 5

Supplemental Table 6

Supplemental Table 7

Supplemental Table 8

Supplemental Table 9

Supplemental Table 10

## Data Availability

The RNA‐seq data and the scRNA‐seq data that support the findings of this study have been deposited in the Gene Expression Omnibus under accession codes GSE165640 and GSE165641, respectively.

## References

[1] R. S. Herbst , D. Morgensztern , C. Boshoff , Nature 2018, 553, 446. 29364287 10.1038/nature25183

[2] R. L. Siegel , K. D. Miller , A. Jemal , CA Cancer J. Clin. 2020, 70, 7. 31912902 10.3322/caac.21590

[3] B. A. Weir , M. S. Woo , G. Getz , S. Perner , L. Ding , R. Beroukhim , W. M. Lin , M. A. Province , A. Kraja , L. A. Johnson , K. Shah , M. Sato , R. K. Thomas , J. A. Barletta , I. B. Borecki , S. Broderick , A. C. Chang , D. Y. Chiang , L. R. Chirieac , J. Cho , Y. Fujii , A. F. Gazdar , T. Giordano , H. Greulich , M. Hanna , B. E. Johnson , M. G. Kris , A. Lash , L. Lin , N. Lindeman , et al., Nature 2007, 450, 893. 17982442 10.1038/nature06358 PMC2538683

[4] F. Skoulidis , J. V. Heymach , Nat. Rev. Cancer 2019, 19, 495. 31406302 10.1038/s41568-019-0179-8 PMC7043073

[5] E. L. Jackson , N. Willis , K. Mercer , R. T. Bronson , D. Crowley , R. Montoya , T. Jacks , D. A. Tuveson , Genes Dev. 2001, 15, 3243. 11751630 10.1101/gad.943001 PMC312845

[6] L. Johnson , K. Mercer , D. Greenbaum , R. T. Bronson , D. Crowley , D. A. Tuveson , T. Jacks , Nature 2001, 410, 1111. 11323676 10.1038/35074129

[7] F. Skoulidis , L. A. Byers , L. Diao , V. A. Papadimitrakopoulou , P. Tong , J. Izzo , C. Behrens , H. Kadara , E. R. Parra , J. R. Canales , J. Zhang , U. Giri , J. Gudikote , M. A. Cortez , C. Yang , Y. Fan , M. Peyton , L. Girard , K. R. Coombes , C. Toniatti , T. P. Heffernan , M. Choi , G. M. Frampton , V. Miller , J. N. Weinstein , R. S. Herbst , K. K. Wong , J. Zhang , P. Sharma , G. B. Mills , et al., Cancer Discovery 2015, 5, 860. 26069186 10.1158/2159-8290.CD-14-1236 PMC4527963

[8] M. A. Gillette , S. Satpathy , S. Cao , S. M. Dhanasekaran , S. V. Vasaikar , K. Krug , F. Petralia , Y. Li , W. W. Liang , B. Reva , A. Krek , J. Ji , X. Song , W. Liu , R. Hong , L. Yao , L. Blumenberg , S. R. Savage , M. C. Wendl , B. Wen , K. Li , L. C. Tang , M. A. MacMullan , S. C. Avanessian , M. H. Kane , C. J. Newton , M. Cornwell , R. B. Kothadia , W. Ma , S. Yoo , R. Mannan , et al., Cell 2020, 182, 200.32649874

[9] Y. J. Chen , T. I. Roumeliotis , Y. H. Chang , C. T. Chen , C. L. Han , M. H. Lin , H. W. Chen , G. C. Chang , Y. L. Chang , C. T. Wu , M. W. Lin , M. S. Hsieh , Y. T. Wang , Y. R. Chen , I. Jonassen , F. Z. Ghavidel , Z. S. Lin , K. T. Lin , C. W. Chen , P. Y. Sheu , C. T. Hung , K. C. Huang , H. C. Yang , P. Y. Lin , T. C. Yen , Y. W. Lin , J. H. Wang , L. Raghav , C. Y. Lin , Y. S. Chen , et al., Cell 2020, 182, 226.32649875

[10] J. Y. Xu , C. Zhang , X. Wang , L. Zhai , Y. Ma , Y. Mao , K. Qian , C. Sun , Z. Liu , S. Jiang , M. Wang , L. Feng , L. Zhao , P. Liu , B. Wang , X. Zhao , H. Xie , X. Yang , L. Zhao , Y. Chang , J. Jia , X. Wang , Y. Zhang , Y. Wang , Y. Yang , Z. Wu , L. Yang , B. Liu , T. Zhao , S. Ren , A. Sun , Y. Zhao , W. Ying , F. Wang , G. Wang , Y. Zhang , S. Cheng , J. Qin , X. Qian , Y. Wang , J. Li , F. He , T. Xiao , M. Tan , Cell 2020, 182, 245. 32649877 10.1016/j.cell.2020.05.043

[11] H. Ji , M. R. Ramsey , D. N. Hayes , C. Fan , K. McNamara , P. Kozlowski , C. Torrice , M. C. Wu , T. Shimamura , S. A. Perera , M. C. Liang , D. Cai , G. N. Naumov , L. Bao , C. M. Contreras , D. Li , L. Chen , J. Krishnamurthy , J. Koivunen , L. R. Chirieac , R. F. Padera , R. T. Bronson , N. I. Lindeman , D. C. Christiani , X. Lin , G. I. Shapiro , P. A. Janne , B. E. Johnson , M. Meyerson , D. J. Kwiatkowski , D. H. Castrillon , N. Bardeesy , N. E. Sharpless , K. K. Wong , Nature 2007, 448, 807. 17676035 10.1038/nature06030

[12] A. Ventura , D. G. Kirsch , M. E. McLaughlin , D. A. Tuveson , J. Grimm , L. Lintault , J. Newman , E. E. Reczek , R. Weissleder , T. Jacks , Nature 2007, 445, 661. 17251932 10.1038/nature05541

[13] D. A. Barbie , P. Tamayo , J. S. Boehm , S. Y. Kim , S. E. Moody , I. F. Dunn , A. C. Schinzel , P. Sandy , E. Meylan , C. Scholl , S. Frohling , E. M. Chan , M. L. Sos , K. Michel , C. Mermel , S. J. Silver , B. A. Weir , J. H. Reiling , Q. Sheng , P. B. Gupta , R. C. Wadlow , H. Le , S. Hoersch , B. S. Wittner , S. Ramaswamy , D. M. Livingston , D. M. Sabatini , M. Meyerson , R. K. Thomas , E. S. Lander , et al., Nature 2009, 462, 108. 19847166 10.1038/nature08460 PMC2783335

[14] Z. Chen , K. Cheng , Z. Walton , Y. Wang , H. Ebi , T. Shimamura , Y. Liu , T. Tupper , J. Ouyang , J. Li , P. Gao , M. S. Woo , C. Xu , M. Yanagita , A. Altabef , S. Wang , C. Lee , Y. Nakada , C. G. Pena , Y. Sun , Y. Franchetti , C. Yao , A. Saur , M. D. Cameron , M. Nishino , D. N. Hayes , M. D. Wilkerson , P. J. Roberts , C. B. Lee , N. Bardeesy , et al., Nature 2012, 483, 613. 22425996 10.1038/nature10937 PMC3385933

[15] F. J. Sanchez‐Rivera , T. Papagiannakopoulos , R. Romero , T. Tammela , M. R. Bauer , A. Bhutkar , N. S. Joshi , L. Subbaraj , R. T. Bronson , W. Xue , T. Jacks , Nature 2014, 516, 428. 25337879 10.1038/nature13906 PMC4292871

[16] H. Sies , C. Berndt , D. P. Jones , Annu. Rev. Biochem. 2017, 86, 715. 28441057 10.1146/annurev-biochem-061516-045037

[17] J. D. Hayes , A. T. Dinkova‐Kostova , K. D. Tew , Cancer Cell 2020, 38, 167. 32649885 10.1016/j.ccell.2020.06.001 PMC7439808

[18] J. E. Klaunig , Curr. Pharm. Des. 2018, 24, 4771. 30767733 10.2174/1381612825666190215121712

[19] V. I. Sayin , M. X. Ibrahim , E. Larsson , J. A. Nilsson , P. Lindahl , M. O. Bergo , Sci. Transl. Med. 2014, 6, 221ra15. 10.1126/scitranslmed.3007653 24477002

[20] G. M. DeNicola , F. A. Karreth , T. J. Humpton , A. Gopinathan , C. Wei , K. Frese , D. Mangal , K. H. Yu , C. J. Yeo , E. S. Calhoun , F. Scrimieri , J. M. Winter , R. H. Hruban , C. Iacobuzio‐Donahue , S. E. Kern , I. A. Blair , D. A. Tuveson , Nature 2011, 475, 106. 21734707 10.1038/nature10189 PMC3404470

[21] S. C. Lu , Biochim. Biophys. Acta 2013, 1830, 3143. 22995213 10.1016/j.bbagen.2012.09.008 PMC3549305

[22] R. Brigelius‐Flohe , M. Maiorino , Biochim. Biophys. Acta 2013, 1830, 3289. 23201771 10.1016/j.bbagen.2012.11.020

[23] W. Ying , Antioxid. Redox Signaling 2008, 10, 179. 10.1089/ars.2007.1672 18020963

[24] R. C. Stanton , IUBMB Life 2012, 64, 362. 22431005 10.1002/iub.1017 PMC3325335

[25] R. A. Cairns , I. S. Harris , T. W. Mak , Nat. Rev. Cancer 2011, 11, 85. 21258394 10.1038/nrc2981

[26] S. L. Blair , P. Heerdt , S. Sachar , A. Abolhoda , S. Hochwald , H. Cheng , M. Burt , Cancer Res. 1997, 57, 152. 8988057

[27] G. K. Balendiran , R. Dabur , D. Fraser , Cell Biochem. Funct. 2004, 22, 343. 15386533 10.1002/cbf.1149

[28] A. Gupta , S. Srivastava , R. Prasad , S. M. Natu , B. Mittal , M. P. Negi , A. N. Srivastava , Respirology 2010, 15, 349. 20199646 10.1111/j.1440-1843.2009.01703.x

[29] E. Y. Bassoy , J. E. Towne , C. Gabay , Immunol. Rev. 2018, 281, 169. 29247994 10.1111/imr.12610

[30] M. F. Neurath , Cytokine Growth Factor Rev. 2020, 55, 70. 32540133 10.1016/j.cytogfr.2020.06.006

[31] L. Zhou , V. Todorovic , Adv. Exp. Med. Biol. 2021, 21, 191. 32026417 10.1007/5584_2020_488

[32] Y. Carrier , H. L. Ma , H. E. Ramon , L. Napierata , C. Small , M. O'Toole , D. A. Young , L. A. Fouser , C. Nickerson‐Nutter , M. Collins , K. Dunussi‐Joannopoulos , Q. G. Medley , J. Invest. Dermatol. 2011, 131, 2428. 21881584 10.1038/jid.2011.234

[33] A. L. Buhl , J. Wenzel , Front. Immunol. 2019, 10, 1162. 31191535 10.3389/fimmu.2019.01162 PMC6545975

[34] M. Catapano , M. Vergnano , M. Romano , S. K. Mahil , S. E. Choon , A. D. Burden , H. S. Young , I. M. Carr , H. J. Lachmann , G. Lombardi , C. H. Smith , F. D. Ciccarelli , J. N. Barker , F. Capon , J. Invest. Dermatol. 2020, 140, 816. 31539532 10.1016/j.jid.2019.08.444 PMC7097848

[35] O. Medina‐Contreras , A. Harusato , H. Nishio , K. L. Flannigan , V. Ngo , G. Leoni , P. A. Neumann , D. Geem , L. N. Lili , R. A. Ramadas , B. Chassaing , A. T. Gewirtz , J. E. Kohlmeier , C. A. Parkos , J. E. Towne , A. Nusrat , T. L. Denning , J. Immunol. 2016, 196, 34. 26590314 10.4049/jimmunol.1501312 PMC4684965

[36] K. Scheibe , I. Backert , S. Wirtz , A. Hueber , G. Schett , M. Vieth , H. C. Probst , T. Bopp , M. F. Neurath , C. Neufert , Gut 2017, 66, 823. 26783184 10.1136/gutjnl-2015-310374

[37] K. Scheibe , C. Kersten , A. Schmied , M. Vieth , T. Primbs , B. Carle , F. Knieling , J. Claussen , A. C. Klimowicz , J. Zheng , P. Baum , S. Meyer , S. Schurmann , O. Friedrich , M. J. Waldner , T. Rath , S. Wirtz , G. Kollias , A. B. Ekici , R. Atreya , E. L. Raymond , M. L. Mbow , M. F. Neurath , C. Neufert , Gastroenterology 2019, 156, 1082. 30452921 10.1053/j.gastro.2018.11.029

[38] S. Gunther , E. J. Sundberg , J. Immunol. 2014, 193, 921. 24935927 10.4049/jimmunol.1400538

[39] S. Marrakchi , P. Guigue , B. R. Renshaw , A. Puel , X. Y. Pei , S. Fraitag , J. Zribi , E. Bal , C. Cluzeau , M. Chrabieh , J. E. Towne , J. Douangpanya , C. Pons , S. Mansour , V. Serre , H. Makni , N. Mahfoudh , F. Fakhfakh , C. Bodemer , J. Feingold , S. Hadj‐Rabia , M. Favre , E. Genin , M. Sahbatou , A. Munnich , J. L. Casanova , J. E. Sims , H. Turki , H. Bachelez , A. Smahi , N. Engl. J. Med. 2011, 365, 620. 21848462 10.1056/NEJMoa1013068

[40] K. Sugiura , A. Takemoto , M. Yamaguchi , H. Takahashi , Y. Shoda , T. Mitsuma , K. Tsuda , E. Nishida , Y. Togawa , K. Nakajima , A. Sakakibara , S. Kawachi , M. Shimizu , Y. Ito , T. Takeichi , M. Kono , Y. Ogawa , Y. Muro , A. Ishida‐Yamamoto , S. Sano , H. Matsue , A. Morita , H. Mizutani , H. Iizuka , M. Muto , M. Akiyama , J. Invest. Dermatol. 2013, 133, 2514. 23698098 10.1038/jid.2013.230

[41] M. F. Neurath , S. Finotto , Cytokine Growth Factor Rev. 2012, 23, 315. 23022528 10.1016/j.cytogfr.2012.08.009

[42] D. Lin , M. Zhang , H. Guo , Y. Deng , B. Zhong , F. Liao , Z. Xu , Acta Biochim. Biophys. Sin. 2020, 52, 691. 32347900 10.1093/abbs/gmaa032

[43] M. Zhang , W. Yang , P. Wang , Y. Deng , Y. T. Dong , F. F. Liu , R. Huang , P. Zhang , Y. Q. Duan , X. D. Liu , D. Lin , Q. Chu , B. Zhong , Nat. Commun. 2020, 11, 6119. 33257678 10.1038/s41467-020-19973-6 PMC7704643

[44] X. Wang , X. Zhao , C. Feng , A. Weinstein , R. Xia , W. Wen , Q. Lv , S. Zuo , P. Tang , X. Yang , X. Chen , H. Wang , S. Zang , L. Stollings , T. L. Denning , J. Jiang , J. Fan , G. Zhang , X. Zhang , Y. Zhu , W. Storkus , B. Lu , Cancer Cell 2015, 28, 296. 26321222 10.1016/j.ccell.2015.07.014 PMC4573903

[45] E. Gentilin , E. Simoni , M. Candito , D. Cazzador , L. Astolfi , Trends Mol. Med. 2019, 25, 1123. 31473143 10.1016/j.molmed.2019.08.002

[46] C. M. Henry , G. P. Sullivan , D. M. Clancy , I. S. Afonina , D. Kulms , S. J. Martin , Cell Rep. 2016, 14, 708. 26776523 10.1016/j.celrep.2015.12.072

[47] G. P. Sullivan , C. M. Henry , D. M. Clancy , T. Mametnabiev , E. Belotcerkovskaya , P. Davidovich , S. Sura‐Trueba , A. V. Garabadzhiu , S. J. Martin , Cell Death Dis. 2018, 9, 378. 29515113 10.1038/s41419-018-0385-4 PMC5841435

[48] H. Bachelez , S. E. Choon , S. Marrakchi , A. D. Burden , T. F. Tsai , A. Morita , H. Turki , D. B. Hall , M. Shear , P. Baum , S. J. Padula , C. Thoma , N. Engl. J. Med. 2019, 380, 981. 30855749 10.1056/NEJMc1811317

[49] K. J. Mackenzie , P. Carroll , C. A. Martin , O. Murina , A. Fluteau , D. J. Simpson , N. Olova , H. Sutcliffe , J. K. Rainger , A. Leitch , R. T. Osborn , A. P. Wheeler , M. Nowotny , N. Gilbert , T. Chandra , M. A. M. Reijns , A. P. Jackson , Nature 2017, 548, 461. 28738408 10.1038/nature23449 PMC5870830

[50] S. F. Bakhoum , L. C. Cantley , Cell 2018, 174, 1347. 30193109 10.1016/j.cell.2018.08.027 PMC6136429

[51] D. Lambrechts , E. Wauters , B. Boeckx , S. Aibar , D. Nittner , O. Burton , A. Bassez , H. Decaluwe , A. Pircher , K. Van den Eynde , B. Weynand , E. Verbeken , P. De Leyn , A. Liston , J. Vansteenkiste , P. Carmeliet , S. Aerts , B. Thienpont , Nat. Med. 2018, 24, 1277. 29988129 10.1038/s41591-018-0096-5

[52] R. Zilionis , C. Engblom , C. Pfirschke , V. Savova , D. Zemmour , H. D. Saatcioglu , I. Krishnan , G. Maroni , C. V. Meyerovitz , C. M. Kerwin , S. Choi , W. G. Richards , A. De Rienzo , D. G. Tenen , R. Bueno , E. Levantini , M. J. Pittet , A. M. Klein , Immunity 2019, 50, 1317. 30979687 10.1016/j.immuni.2019.03.009 PMC6620049

[53] M. J. Morgan , Z. G. Liu , Cell Res. 2011, 21, 103. 21187859 10.1038/cr.2010.178 PMC3193400

[54] M. Rojo de la Vega , E. Chapman , D. D. Zhang , Cancer Cell 2018, 34, 21. 29731393 10.1016/j.ccell.2018.03.022 PMC6039250

[55] C. Wiel , K. L. e. Gal , M. X. Ibrahim , C. A. Jahangir , M. Kashif , H. Yao , D. V. Ziegler , X. Xu , T. Ghosh , T. Mondal , C. Kanduri , P. Lindahl , V. I. Sayin , M. O. Bergo , Cell 2019, 178, 330. 31257027 10.1016/j.cell.2019.06.005

[56] L. O. Klotz , C. Sanchez‐Ramos , I. Prieto‐Arroyo , P. Urbanek , H. Steinbrenner , M. Monsalve , Redox Biol. 2015, 6, 51. 26184557 10.1016/j.redox.2015.06.019 PMC4511623

[57] J. St‐Pierre , S. Drori , M. Uldry , J. M. Silvaggi , J. Rhee , S. Jager , C. Handschin , K. Zheng , J. Lin , W. Yang , D. K. Simon , R. Bachoo , B. M. Spiegelman , Cell 2006, 127, 397. 17055439 10.1016/j.cell.2006.09.024

[58] H. Lu , D. Samanta , L. Xiang , H. Zhang , H. Hu , I. Chen , J. W. Bullen , G. L. Semenza , Proc. Natl. Acad. Sci. U. S. A. 2015, 112, E4600. 26229077 10.1073/pnas.1513433112 PMC4547233

[59] P. Bouwman , J. Jonkers , Nat. Rev. Cancer 2012, 12, 587. 22918414 10.1038/nrc3342

[60] H. Yang , R. M. Villani , H. Wang , M. J. Simpson , M. S. Roberts , M. Tang , X. Liang , J. Exp. Clin. Cancer Res. 2018, 37, 266. 30382874 10.1186/s13046-018-0909-x PMC6211502

[61] R. X. Huang , P. K. Zhou , Signal Transduction Targeted Ther. 2020, 5, 60. 10.1038/s41392-020-0150-x PMC7192953 32355263

[62] D. M. Pardoll , Nat. Rev. Cancer 2012, 12, 252. 22437870 10.1038/nrc3239 PMC4856023

[63] W. Wang , M. Green , J. E. Choi , M. Gijon , P. D. Kennedy , J. K. Johnson , P. Liao , X. Lang , I. Kryczek , A. Sell , H. Xia , J. Zhou , G. Li , J. Li , W. Li , S. Wei , L. Vatan , H. Zhang , W. Szeliga , W. Gu , R. Liu , T. S. Lawrence , C. Lamb , Y. Tanno , M. Cieslik , E. Stone , G. Georgiou , T. A. Chan , A. Chinnaiyan , W. Zou , Nature 2019, 569, 270. 31043744 10.1038/s41586-019-1170-y PMC6533917

[64] X.‐M. Wang , C. i. Yang , Y. Zhao , Z.‐G. Xu , W. Yang , P. Wang , D. Lin , B. Xiong , J.‐Y. Fang , C. Dong , A. B. Zhong , Nat. Cancer 2020, 1, 811. 10.1038/s43018-020-0089-4 35122046

[65] Y. Zhao , X. Wang , Q. Wang , Y. Deng , K. Li , M. Zhang , Q. Zhang , J. Zhou , H. Y. Wang , P. Bai , Y. Ren , N. Zhang , W. Li , Y. Cheng , W. Xiao , H. N. Du , X. Cheng , L. Yin , X. Fu , D. Lin , Q. Zhou , B. Zhong , Cell Rep. 2018, 22, 2442. 29490279 10.1016/j.celrep.2018.02.007

[66] Z. Cai , M. X. Zhang , Z. Tang , Q. Zhang , J. Ye , T. C. Xiong , Z. D. Zhang , B. Zhong , J. Exp. Med. 2020, 217, e20191174. 32130408 10.1084/jem.20191174 PMC7201923

[67] Q. Zhang , Z. Tang , R. An , L. Ye , B. Zhong , Cell Res. 2020, 30, 914. 32457395 10.1038/s41422-020-0341-6 PMC7608407

[68] T. Liuyu , K. Yu , L. Ye , Z. Zhang , M. Zhang , Y. Ren , Z. Cai , Q. Zhu , D. Lin , B. Zhong , Cell Res. 2019, 29, 67. 30410068 10.1038/s41422-018-0107-6 PMC6318273

