# Supplementary material for: IL‐36γ and IL‐36Ra Reciprocally Regulate NSCLC Progression by Modulating GSH Homeostasis and Oxidative Stress‐Induced Cell Death
Source: Adv Sci (Weinh). 2021 Aug 8;8(19):2101501. doi: 10.1002/advs.202101501 (PMC8498882; doi:10.1002/advs.202101501)
Supplement: Supplementary file 1 — Supporting Information [file ADVS-8-2101501-s008.pdf]

© 2021 Wiley-VCH GmbH

ADVANCED SCIENCE
Open Access

Supporting Information

for *Adv. Sci.*, DOI: 10.1002/advs.202101501

IL-36γ and IL-36Ra Reciprocally Regulate NSCLC Progression by Modulating GSH Homeostasis and Oxidative Stress-Induced Cell Death

Peng Wang, Wei Yang, Hao Guo, Hong-Peng Dong, Yu-Yao Guo, Hu Gan, Zou Wang, Yongbo Cheng, Yu Deng, Shizhe Xie, Xinglou Yang, Dandan Lin, and Bo Zhong**

Figure S1

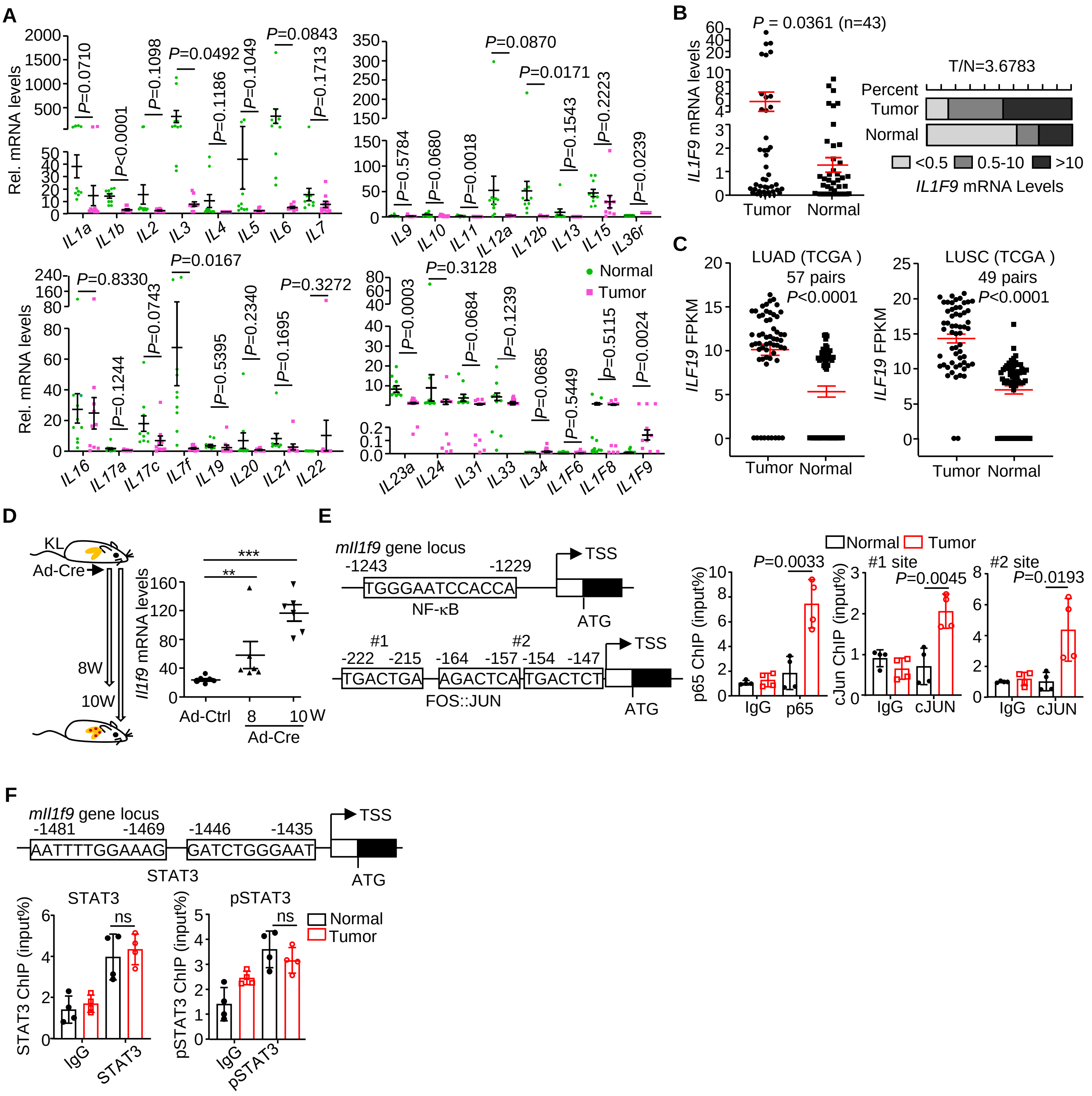
A
Rel. mRNA levels
Normal
Tumor
B
IL1F9 mRNA levels
P = 0.0361 (n=43)
Tumor
Normal
T/N=3.6783
Percent
<0.5
0.5-10
>10
IL1F9 mRNA Levels
C
LUAD (TCGA)
57 pairs
P<0.0001
LUSC (TCGA)
49 pairs
ILF19 FPKM
D
KL
Ad-Cre
8W
10W
Il1f9 mRNA levels
Ad-Ctrl
E
mIl1f9 gene locus
TGGGAATCCACCA
NF-κB
TSS
ATG
TGACTGA
AGACTCA
TGACTCT
FOS::JUN
p65 ChIP (input%)
cJun ChIP (input%)
#1 site
#2 site
F
AATTTTGGAAAG
GATCTGGGAAT
STAT3
pSTAT3
STAT3 ChIP (input%)
pSTAT3 ChIP (input%)

Figure S1 *IL1F9* is highly expressed in NSCLC tumor tissues.

(A) qRT-PCR analysis of genes encoding interleukins in the tumor tissues and the adjacent normal tissues of NSCLC patients (n=10).

(B) qRT-PCR analysis of IL1F9 mRNA levels in the tumor tissues and the adjacent normal tissues of NSCLC patients (n=43).

(C) The *IL1F9* expression levels in tumor and their paired non-cancerous adjacent tissues from 57 LUAD patients (left) or 49 LUAD patients (right) from TCGA database.

(D) A scheme of NSCLC induction in KL mice (left) and qRT-PCR analysis of *Il1f9* mRNA levels in tumor-burdened lungs of KL mice infected with Ad-Ctrl (n=6 mice) or Ad-Cre (n=6 mice for 6, 8, or 10 weeks, respectively) (right).

(G-H) Sequence analysis of the mouse *Il1f9* promoter (left) and chromatin immunoprecipitation (ChIP) assay (right) of the binding of p65 and cJun (G) or STAT3 and pSTAT3 (H) on the *Il1f9* promoter in single-cell suspensions prepared from normal lungs or lung tumors of KL mice infected with Ad-Vec or Ad-Cre (n=4 mice) for 10 weeks.

Graphs show mean ± SEM (A-F). Two-tailed student's *t*-test (A-F). Data are representative of two independent experiments (D-F).

Figure S2

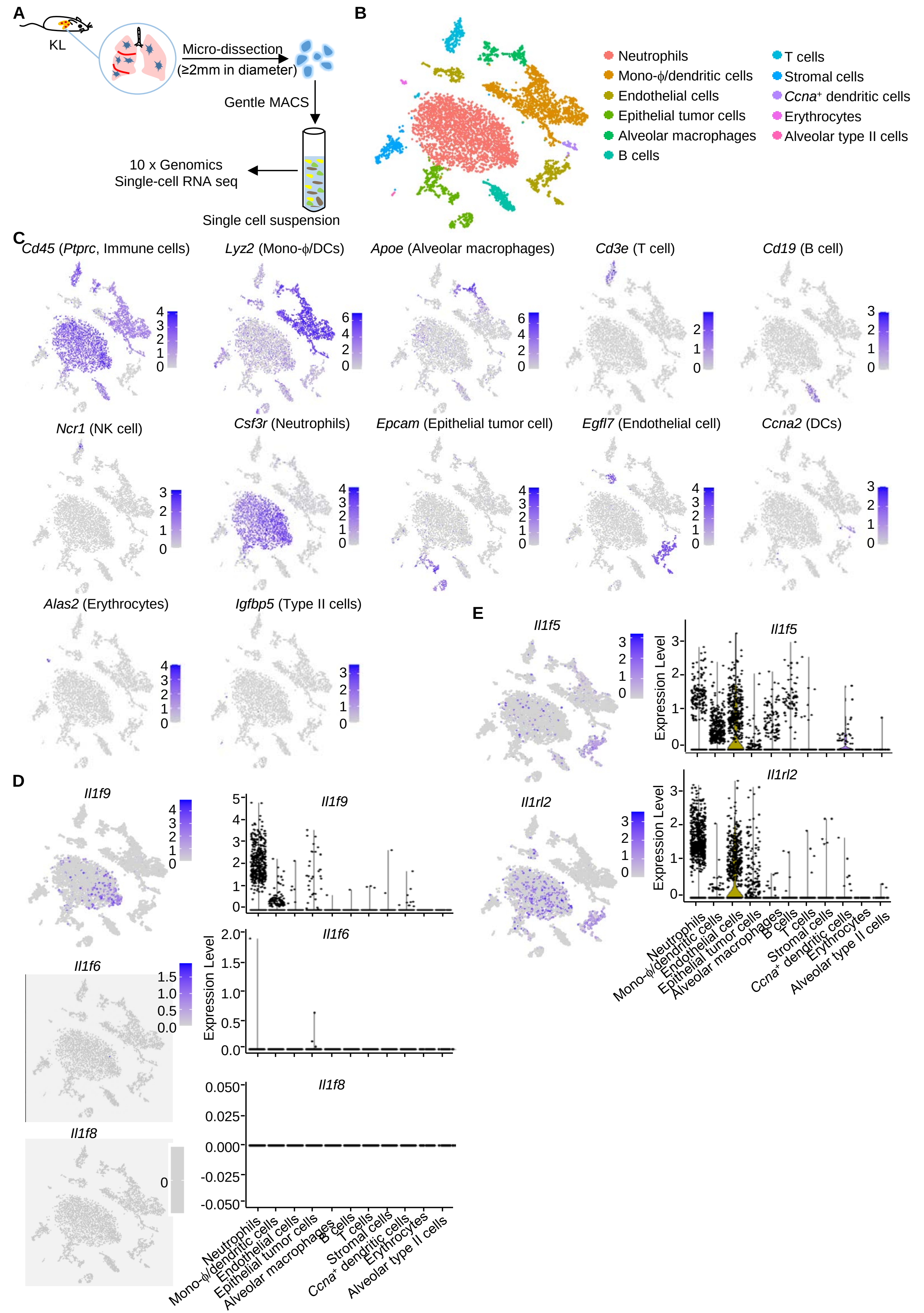
A
KL
Micro-dissection
(≥2mm in diameter)
Gentle MACS
10 x Genomics
Single-cell RNA seq
Single cell suspension
B
Neutrophils
Mono-ϕ/dendritic cells
Endothelial cells
Epithelial tumor cells
Alveolar macrophages
B cells
T cells
Stromal cells
Ccna+ dendritic cells
Erythrocytes
Alveolar type II cells
C
Cd45 (Ptprc, Immune cells)
Lyz2 (Mono-ϕ/DCs)
Apoe (Alveolar macrophages)
Cd3e (T cell)
Cd19 (B cell)
Ncr1 (NK cell)
Csf3r (Neutrophils)
Epcam (Epithelial tumor cell)
Egfl7 (Endothelial cell)
Ccna2 (DCs)
Alas2 (Erythrocytes)
Igfbp5 (Type II cells)
D
Il1f9
Il1f6
Il1f8
Expression Level
E
Il1f5
Il1rl2
Expression Level

Figure S2 Single-cell transcriptional profiling of lung tumors from KL mice.

(A) A scheme of experimental workflow for 10x genomics scRNA-seq of lung tumors from KL mice (n=2) intranasally injected with Ad-Cre for 10 weeks.

(B) t-SNE plots of the 7180 cells with annotated cell types. Colored dots represent cells expressing different marker genes.

(C) t-SNE plot of cells expressing the signature genes.

(D) t-SNE plot (left) and violin dots graph (right) of *Il1f9*, *Il1f6* and *Il1f8* in the 7180 cells of KL lung tumor.

(E) t-SNE plot (left) and violin dots graph (right) of *Il1f5* and *Il1rl2* in the 7180 cells of KL lung tumor.

Figure S3

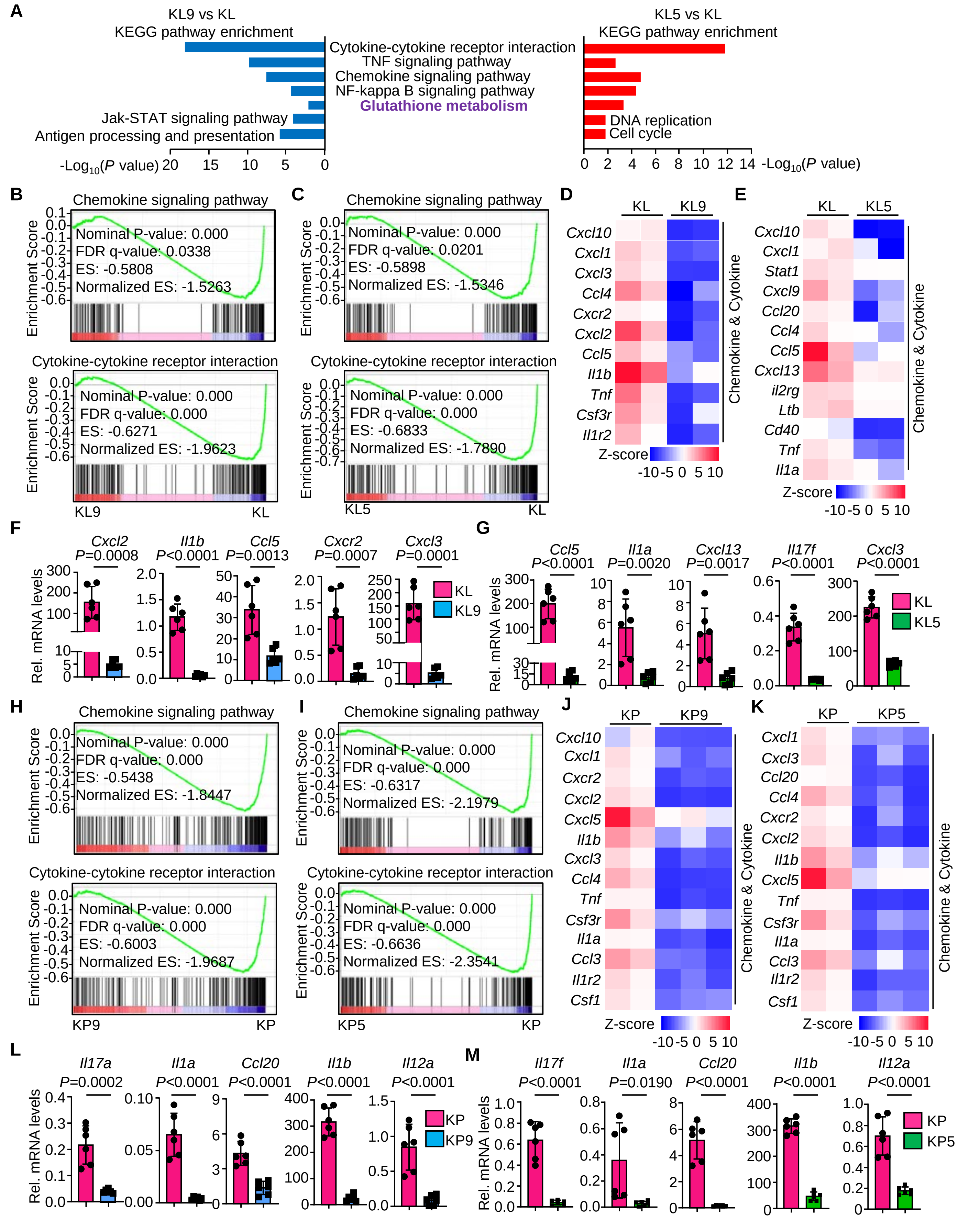
A
KL9 vs KL
KEGG pathway enrichment
KL5 vs KL
KEGG pathway enrichment
Cytokine-cytokine receptor interaction
TNF signaling pathway
Chemokine signaling pathway
NF-kappa B signaling pathway
Glutathione metabolism
Jak-STAT signaling pathway
Antigen processing and presentation
DNA replication
Cell cycle
-Log10(P value)
B
C
Chemokine signaling pathway
Cytokine-cytokine receptor interaction
Enrichment Score
Nominal P-value: 0.000
FDR q-value: 0.0338
ES: -0.5808
Normalized ES: -1.5263
FDR q-value: 0.000
ES: -0.6271
Normalized ES: -1.9623
FDR q-value: 0.0201
ES: -0.5898
Normalized ES: -1.5346
ES: -0.6833
Normalized ES: -1.7890
KL9
KL
KL5
D
E
Chemokine & Cytokine
Z-score
F
G
Rel. mRNA levels
Cxcl2 P=0.0008
Il1b P<0.0001
Ccl5 P=0.0013
Cxcr2 P=0.0007
Cxcl3 P=0.0001
Ccl5 P<0.0001
Il1a P=0.0020
Cxcl13 P=0.0017
Il17f P<0.0001
Cxcl3 P<0.0001
H
I
J
K
ES: -0.5438
Normalized ES: -1.8447
ES: -0.6317
Normalized ES: -2.1979
ES: -0.6003
Normalized ES: -1.9687
ES: -0.6636
Normalized ES: -2.3541
KP9
KP
KP5
L
M
Il17a P=0.0002
Il1a P<0.0001
Ccl20 P<0.0001
Il1b P<0.0001
Il12a P<0.0001
Il17f P<0.0001
Il1a P=0.0190
Ccl20 P<0.0001
Il1b P<0.0001
Il12a P<0.0001

Figure S3 Knockout of IL-36γ or IL-36Ra downregulates expression of cytokines and chemokines in NSCLC tumor tissues.

(A) KEGG pathway analysis of genes differentially expressed in tumors from KL and KL9 (left) or KL and KL5 (right) mice that were intranasally injected with Ad-Cre for 10 or 8 weeks, respectively.

(B-C) GSEA plot of genes involved in cytokine and chemokine signaling pathway from the transcriptome data obtained from KL (n=2) and KL9 (n=2) mice (B) or KL (n=2) and KL5 (n=2) mice (C) treated as in (A).

(D-E) Heatmap of the z-scores of the indicated genes from the transcriptome data obtained from KL (n=2) and KL9 (n=2) mice (D) or KL (n=2) and KL5 (n=2) mice (E) treated as in (A).

(F-G) qRT-PCR analysis of the indicated genes in lung tumors isolated from KL (n=6) and KL9 (n=6) mice that were intranasally injected with Ad-Cre for 10 weeks (F) or from KL (n=6) and KL5 (n=6) mice that were injected with Ad-Cre for 8 weeks (G).

(H-I) GSEA plot of genes involved in cytokine and chemokine signaling pathway from transcriptome data obtained from KP (n=2) and KP9 (n=3) mice (H) or KP (n=2) and KP5 (n=3) mice (I) that were intranasally injected with Ad-Cre for 10 weeks or 8 weeks, respectively.

(J-K) Heatmap of the z-scores of the indicated genes from the transcriptome analysis of lung tumors from KP (n=2) and KP9 (n=3) (J) or KP (n=2) and KP5 (n=3) mice (K) treated as in (H-I).

(L-M) qRT-PCR analysis of the indicated genes in lung tumors isolated from KP (n=6) and KP9 (n=6) mice that were intranasally injected with Ad-Cre for 10 weeks (L) or from KP (n=6) and KP5 (n=6) mice that were injected with Ad-Cre for 8 weeks (M).

Graphs show mean ± SEM (F, G, L, M). Two-tailed student's *t*-test (F, G, L, M). Scale bar represents 50 μm (E). Data are representative of two independent experiments (F, G, L, M).

Figure S4

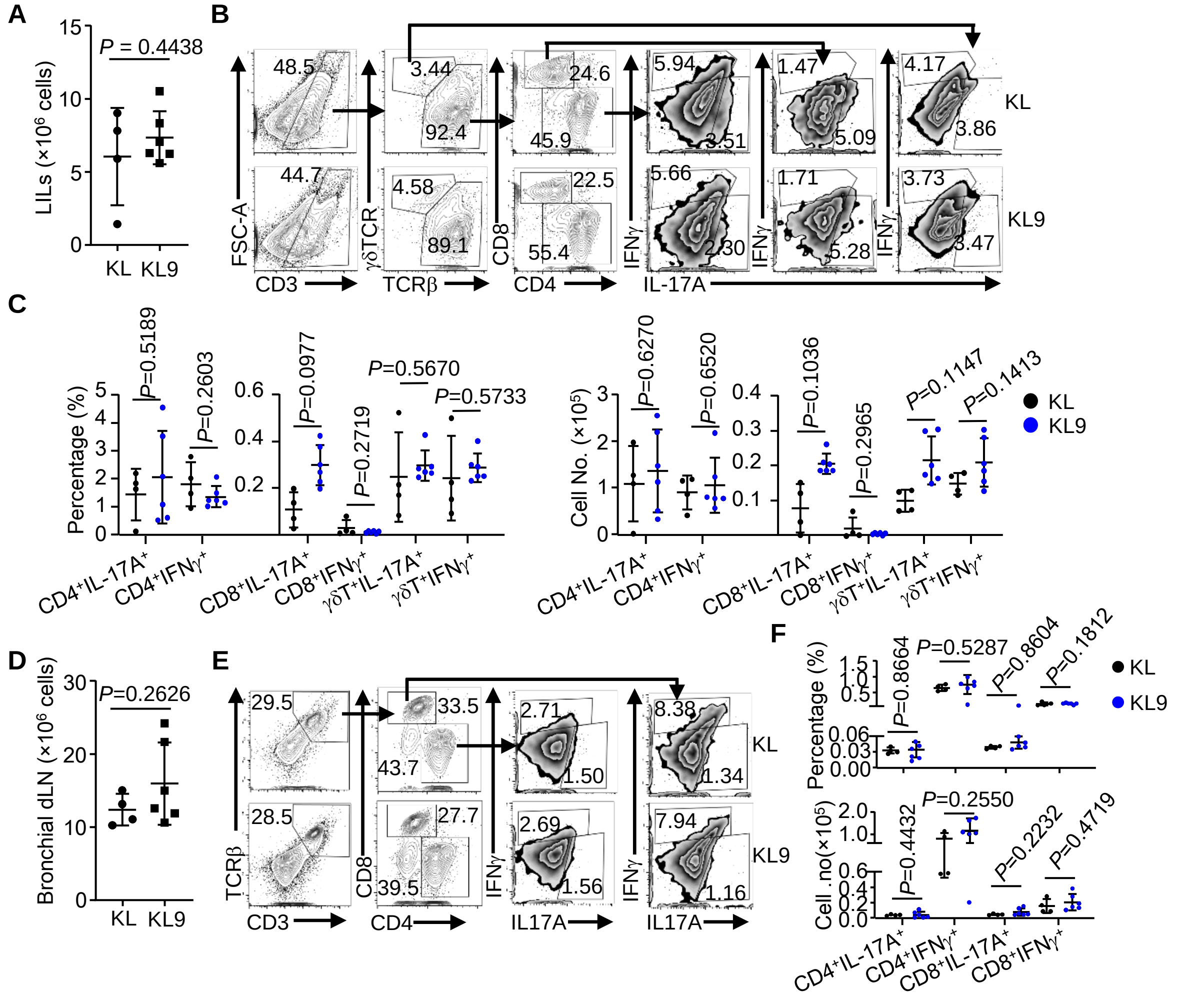

Figure S4 Knockout of IL-36γ did not affect lymphocyte differentiation or expansion in tumor-burdened lungs or in bronchial draining lymph nodes.

(A) Cell numbers of lung infiltrated lymphocytes (LILs) in the tumor-burdened lungs of KL (n =4) and KL9 (n = 6) mice intranasally injected with Ad-Cre (2 x 10^6) for 10 weeks.

(B-C) Flow charts (B) and quantification analysis (C) of LILs from KL (n=4) and KL9 (n=6) mice treated as in (A). The cells were stimulated with PMA and inomycin in the presence of Golgi-stop for 4 h followed by surface and intracellular staining with the indicated antibodies and flow cytometry analysis.

(D) Cell numbers of bronchial draining lymph nodes (dLNs) of KL (n =4) and KL9 (n = 6) mice treated as in (A).

Graphs show mean ± SEM (A, C, D, F). Two-tailed student's *t*-test (A, C, D, F). Data are representative results of two independent experiments (A-F).

Figure S5

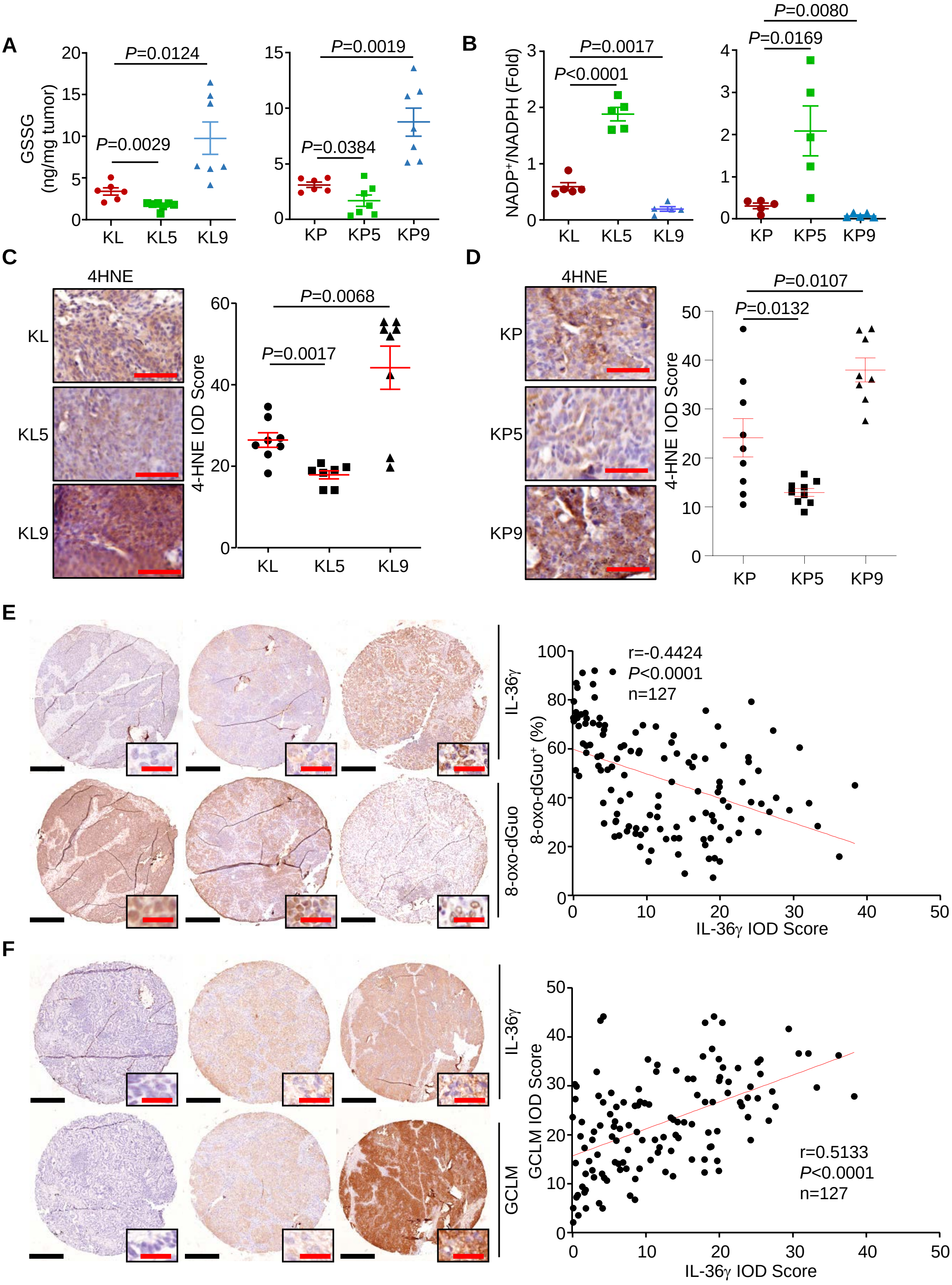
A
GSSG (ng/mg tumor)
P=0.0124
P=0.0029
KL KL5 KL9
P=0.0019
P=0.0384
KP KP5 KP9
B
NADP+/NADPH (Fold)
P=0.0017
P<0.0001
KL KL5 KL9
P=0.0080
P=0.0169
KP KP5 KP9
C
4HNE
KL
KL5
KL9
4-HNE IOD Score
P=0.0068
P=0.0017
D
4HNE
KP
KP5
KP9
4-HNE IOD Score
P=0.0107
P=0.0132
E
IL-36γ
8-oxo-dGuo
8-oxo-dGuo+ (%)
r=-0.4424
P<0.0001
n=127
IL-36γ IOD Score
F
IL-36γ
GCLM
GCLM IOD Score
r=0.5133
P<0.0001
n=127
IL-36γ IOD Score

Figure S5 Knockout of IL-36γ or IL-36Ra alters the redox status in NSCLC tumors.

(A-B) GSSG levels (A) or ratios of $NADP^+$/NADPH in NSCLC tumors from KL (n=6), KL5 (n=7) and KL9 (n=7), or KP (n=6), KP5 (n=7) and KP9 (n=7) mice that were intranasally injected with Ad-Cre ($2x10^6$ pfu) for 10 weeks.

(C-D) Images (left) and quantification analysis (right) of 4HNE staining in the lung tumors from KL (n=8), KL5 (n=7) and KL9 (n=8) mice (C) or KP (n=9), KP5 (n=9) and KP9 (n=8) mice (D) that were intranasally injected with Ad-Cre for 10 weeks.

(E-F) Images (left) and Pearson correlation analysis (right) of IL-36γ and 8-oxo-dGuo staining (E) or IL-36γ and GCLM staining (F) of the tumor biopsies from NSCLC patients (n=127).

Graphs show mean ± SEM (A, B). Two-tailed student's *t*-test (A, B). Scale bars represent 50 μm (C, D), 500 μm (E, F, black) or 10 μm (E, F, red), respectively. Data are representative results of two independent experiments (A-D).

Figure S6

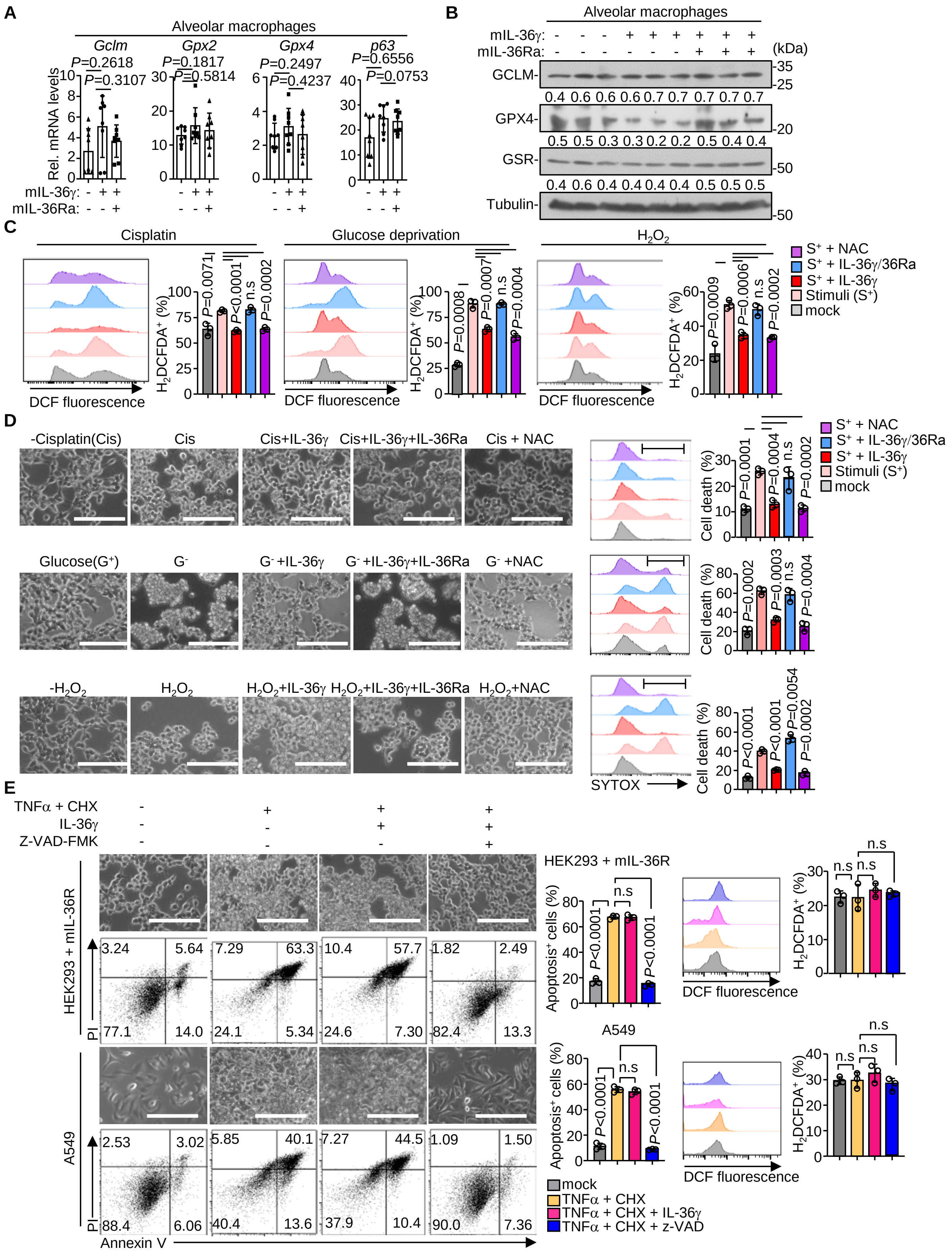
A
Alveolar macrophages
Gclm
Gpx2
Gpx4
p63
P=0.2618
P=0.3107
P=0.1817
P=0.5814
P=0.2497
P=0.4237
P=0.6556
P=0.0753
Rel. mRNA levels
mIL-36γ:
mIL-36Ra:
B
Alveolar macrophages
mIL-36γ:
mIL-36Ra:
(kDa)
GCLM-
0.4 0.6 0.6 0.6 0.7 0.7 0.7 0.7 0.7
GPX4-
0.5 0.5 0.3 0.3 0.2 0.2 0.5 0.4 0.4
GSR-
0.4 0.6 0.4 0.4 0.4 0.4 0.5 0.5 0.5
Tubulin-
C
Cisplatin
Glucose deprivation
H2O2
DCF fluorescence
H2DCFDA+ (%)
P=0.0071
P<0.0001
n.s
P=0.0002
P=0.0008
P=0.0007
n.s
P=0.0004
P=0.0009
P=0.0006
n.s
P=0.0002
S+ + NAC
S+ + IL-36γ/36Ra
S+ + IL-36γ
Stimuli (S+)
mock
D
-Cisplatin(Cis)
Cis
Cis+IL-36γ
Cis+IL-36γ+IL-36Ra
Cis + NAC
Glucose(G+)
G-
G- +IL-36γ
G- +IL-36γ+IL-36Ra
G- +NAC
-H2O2
H2O2
H2O2+IL-36γ
H2O2+IL-36γ+IL-36Ra
H2O2+NAC
SYTOX
Cell death (%)
P=0.0001
P=0.0004
n.s
P=0.0002
P=0.0002
P=0.0003
n.s
P=0.0004
P<0.0001
P<0.0001
P=0.0054
P=0.0002
S+ + NAC
S+ + IL-36γ/36Ra
S+ + IL-36γ
Stimuli (S+)
mock
E
TNFα + CHX
IL-36γ
Z-VAD-FMK
HEK293 + mIL-36R
3.24
5.64
77.1
14.0
7.29
63.3
24.1
5.34
10.4
57.7
24.6
7.30
1.82
2.49
82.4
13.3
PI
A549
2.53
3.02
88.4
6.06
5.85
40.1
40.4
13.6
7.27
44.5
37.9
10.4
1.09
1.50
90.0
7.36
Annexin V
HEK293 + mIL-36R
A549
Apoptosis+ cells (%)
P<0.0001
n.s
DCF fluorescence
H2DCFDA+ (%)
mock
TNFα + CHX
TNFα + CHX + IL-36γ
TNFα + CHX + z-VAD

Figure S6 IL-36γ rescues oxidative stress-induced cell death.

(A) qRT-PCR analysis of *Gclm*, *Gpx2*, *Gpx4*, and *p63* in alveolar macrophages that were stimulated with IL-36γ (20 ng/ml) or IL-36γ plus IL-36Ra (20 ng/ml) for 6 h.

(B) Immunoblot analysis of GCLM, GPX4 and GSR in alveolar macrophages that were unstimulated or stimulated with IL-36γ (20 ng/ml) in the presence or absence of IL-36Ra (20 ng/ml) for 8 h.

(C) Flow cytometry and quantification analysis of H_2DCFDA staining in HEK293-mIL-36R cells that were treated with cisplatin (Cis, 20 μM) for 12 h followed by stimulation with mouse IL-36γ (20 ng/ml), IL-36γ plus IL-36Ra (20 ng/ml), or IL-36γ plus NAC (5 mM) for 8 h (left), cultured in glucose-free DMEM (G^-) for 16 h followed by stimulation with mouse IL-36γ (20 ng/ml), IL-36γ plus IL-36Ra (20 ng/ml), or IL-36γ plus NAC (5 mM) for 8 h (middle), or stimulated with mouse IL-36γ (20 ng/ml), IL-36γ plus IL-36Ra (20 ng/ml), or IL-36γ plus NAC (5 mM) for 7 h followed by H_2O_2 (16 mM) treatment for 1 h (right) (n=3 technical replicates).

(D) Images (left) and flow cytometry and quantification analysis of SYTOX Green staining (right) of HEK293-mIL-36R treated as in (C) (n=3 technical replicates).

(E) Images, flow charts and quantification analysis of HEK293-mIL-36R or A549 cells that were unstimulated or stimulated with CHX (33.3 μg/ml) and TNFα (20 ng/ml) for 18 h followed by treatment with mouse or human IL-36γ (20 ng/ml), Z-VAD-FMK (50 μM) for 8 h followed by Annexin V and PI staining or H_2DCFDA staining (n=3 technical replicates).

Graphs show mean ± SEM (A, C-E). Two-tailed student's *t*-test (A, C-E). Scale bars represent 100 μm (D, E). Data are representative results of two independent experiments.

Figure S7

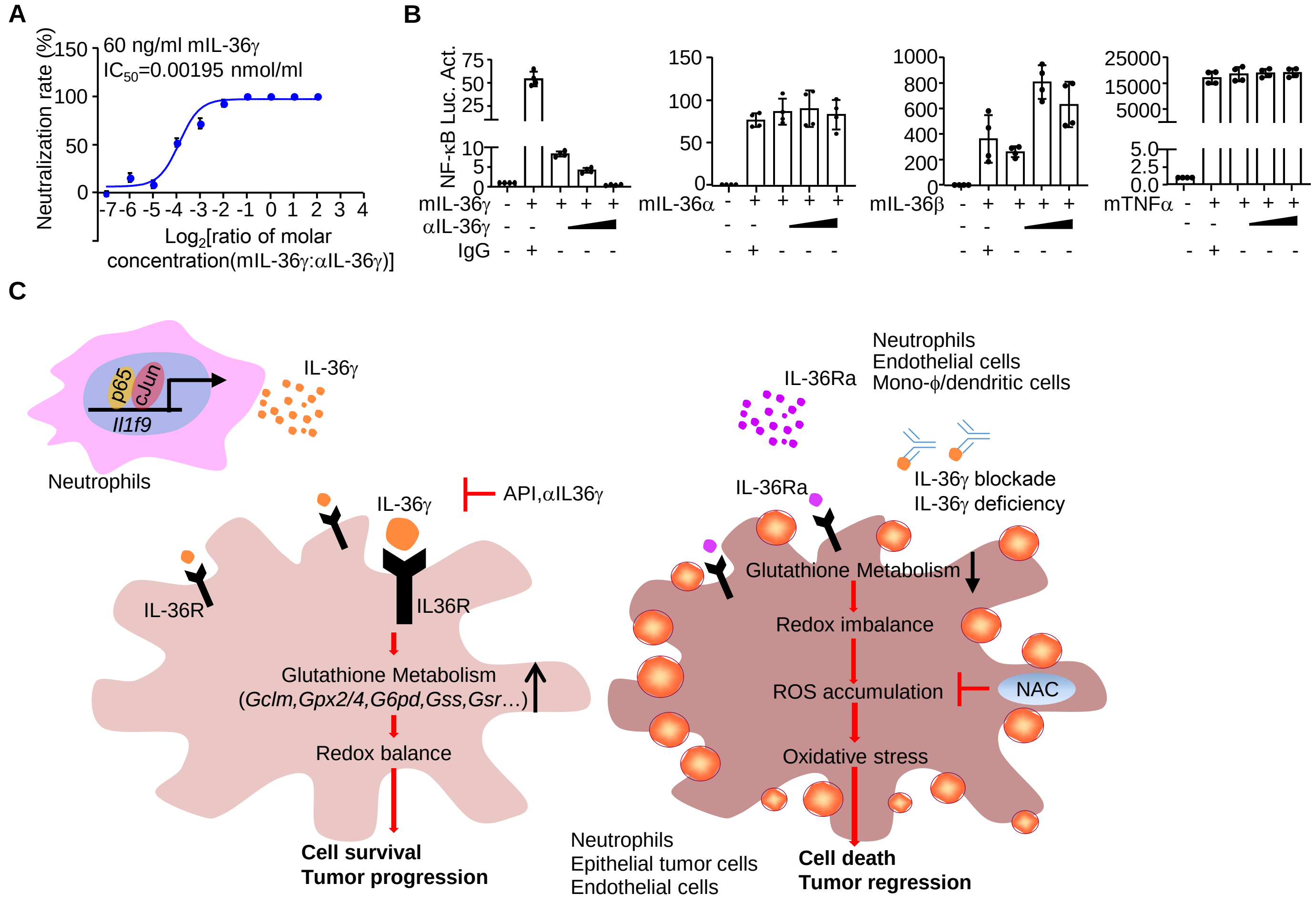

Figure S7 Sensitivity and specificity of the generated anti-IL-36γ antibody.

(A) IC_{50} plot of the anti-IL-36γ in the presence of 60 ng/ml mIL-36γ.

(B) Luciferase reporter assay of HEK293-mIL-36R cells that were transfected with NF-κB luciferase reporter and TK luciferase reporter for 20 h followed by stimulation with mIL-36α, mIL-36β, mIL-36γ or mTNFα in the presence of control IgG (n=4 technical replicates) or anti-IL-36γ (n=4 technical replicates) for 8 h.

(C) A model onIL-36γ- and IL-36Ra-mediated regulation of non-small cell lung cancer progression.

Graphs show mean ± SEM (B). Two-tailed student's *t*-test (B). Data are representative results of two independent experiments (A, B).
